# Supplementary material for: Visible-Light-Mediated Synthesis of Anomeric S-Aryl Glycosides via Electron Donor–Acceptor Complex Using Thianthrenium Salts
Source: Molecules. 2025 Mar 14;30(6):1315. doi: 10.3390/molecules30061315 (PMC11946794; doi:10.3390/molecules30061315)
Supplement: Supplementary file 1 [file molecules-30-01315-s001.zip › molecules-3495983-supplementary.pdf]

Supporting Information

Visible-Light-Mediated Synthesis of Anomeric *S*-Aryl Glycosides via Electron Donor–Acceptor Complex Using Thianthrenium Salts

Zhuoyi Zhou[#1], Yufeng Zhang[#1], Zhiqiang Yu[1], Yuping Liu[1], Zhen Wang[1], Qingju Zhang*[1], Liming Wang*[1]

[1]National Research Centre for Carbohydrate Synthesis, College of Chemistry and Materials of Jiangxi Normal University, 99 Ziyang Avenue, Nanchang, 330022, China.

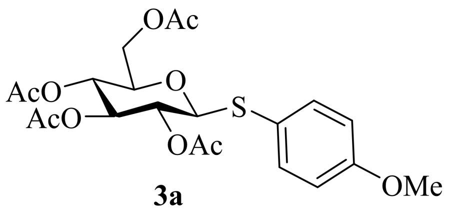
OAc
AcO
AcO
O
S
OAc
OMe

3a

^{1}H-NMR

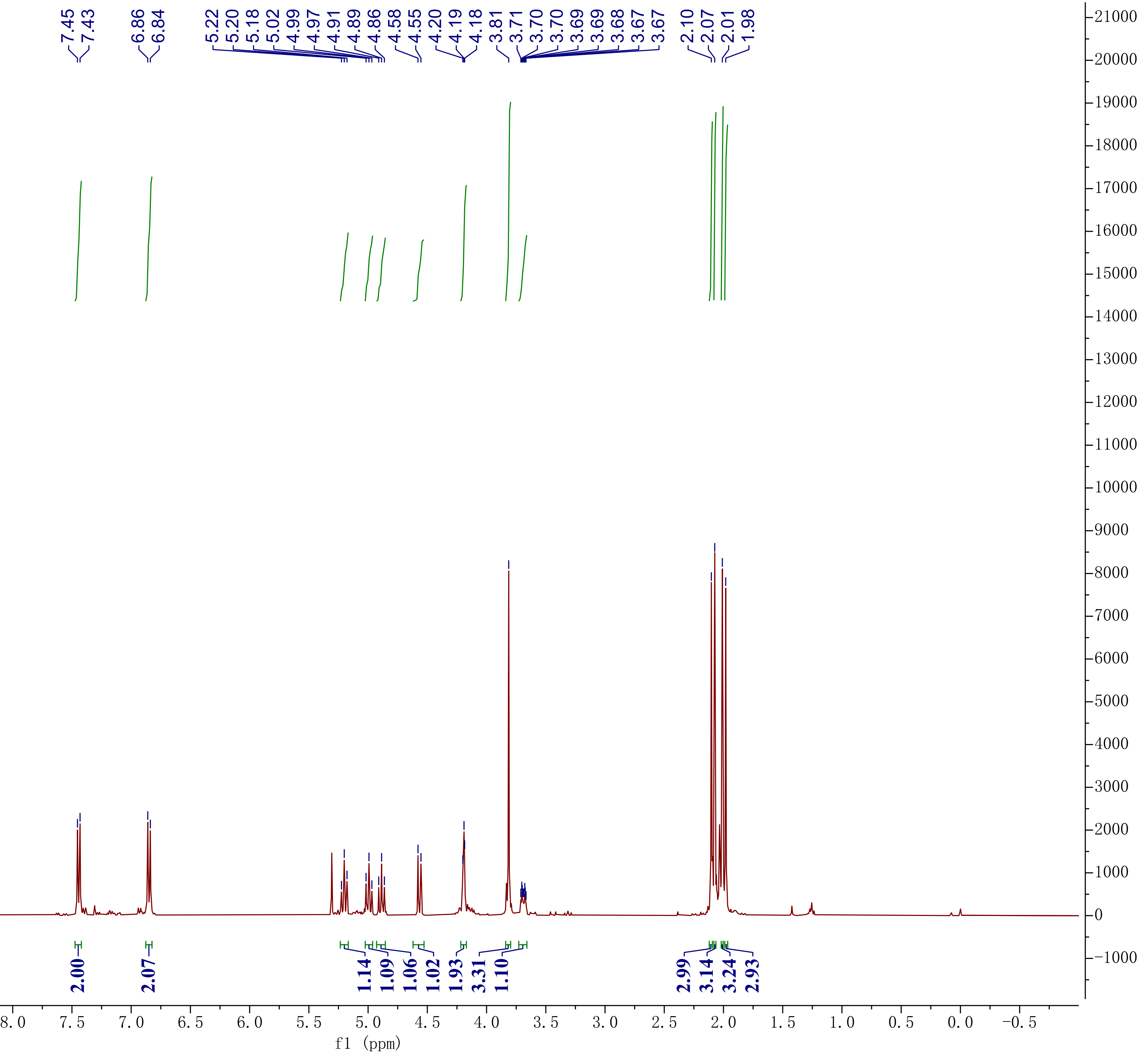
7.45
7.43
6.86
6.84
5.22
5.20
5.18
5.02
4.99
4.97
4.91
4.89
4.86
4.58
4.55
4.20
4.19
4.18
3.81
3.71
3.70
3.70
3.69
3.69
3.68
3.67
3.67
2.10
2.07
2.01
1.98
2.00
2.07
1.14
1.09
1.06
1.02
1.93
3.31
1.10
2.99
3.14
3.24
2.93
f1 (ppm)

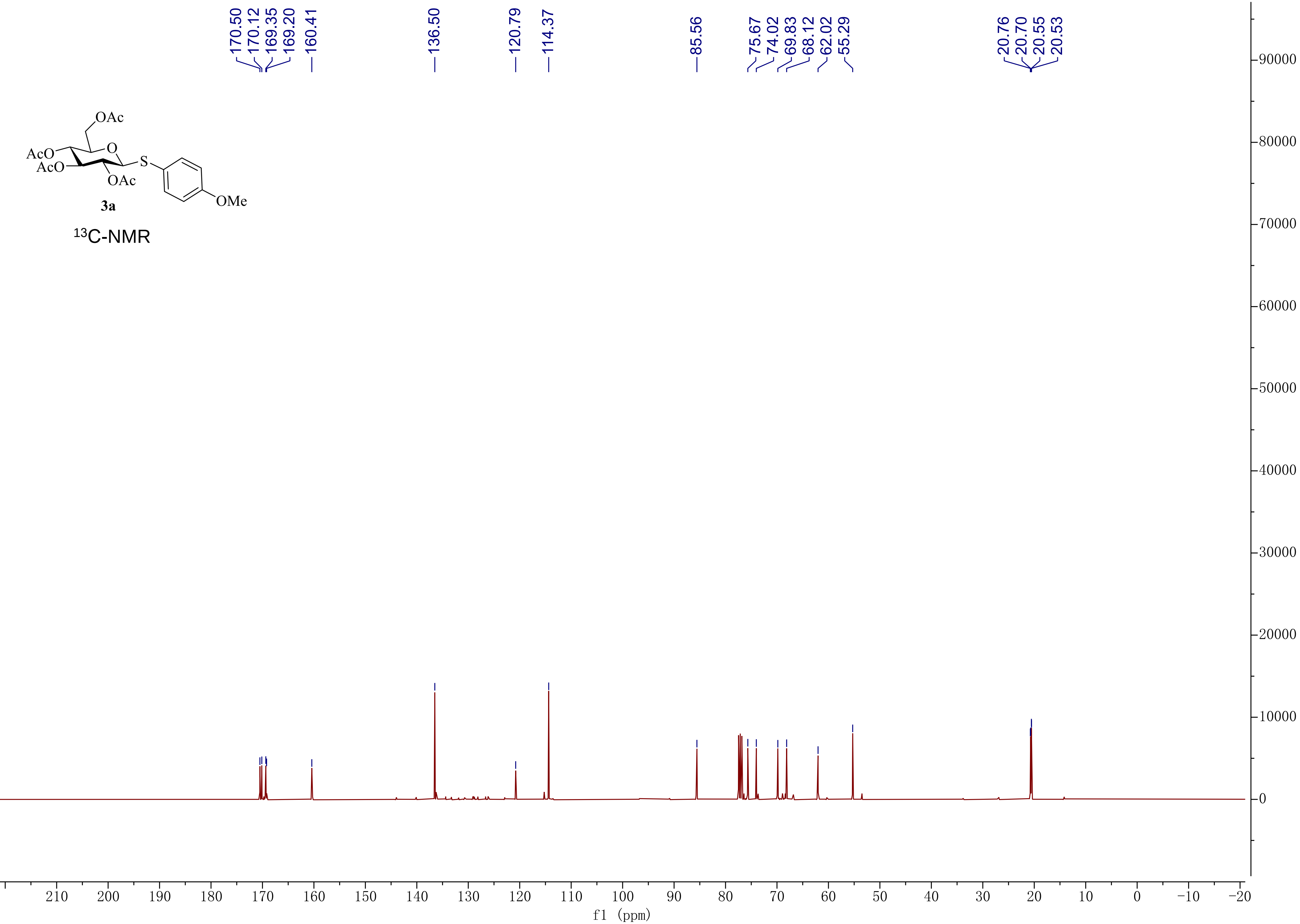
170.50
170.12
169.35
169.20
160.41
136.50
120.79
114.37
85.56
75.67
74.02
69.83
68.12
62.02
55.29
20.76
20.70
20.55
20.53
OAc
AcO
AcO
O
S
OAc
OMe
3a
13C-NMR
90000
80000
70000
60000
50000
40000
30000
20000
10000
0
210 200 190 180 170 160 150 140 130 120 110 100 90 80 70 60 50 40 30 20 10 0 −10 −20
f1 (ppm)

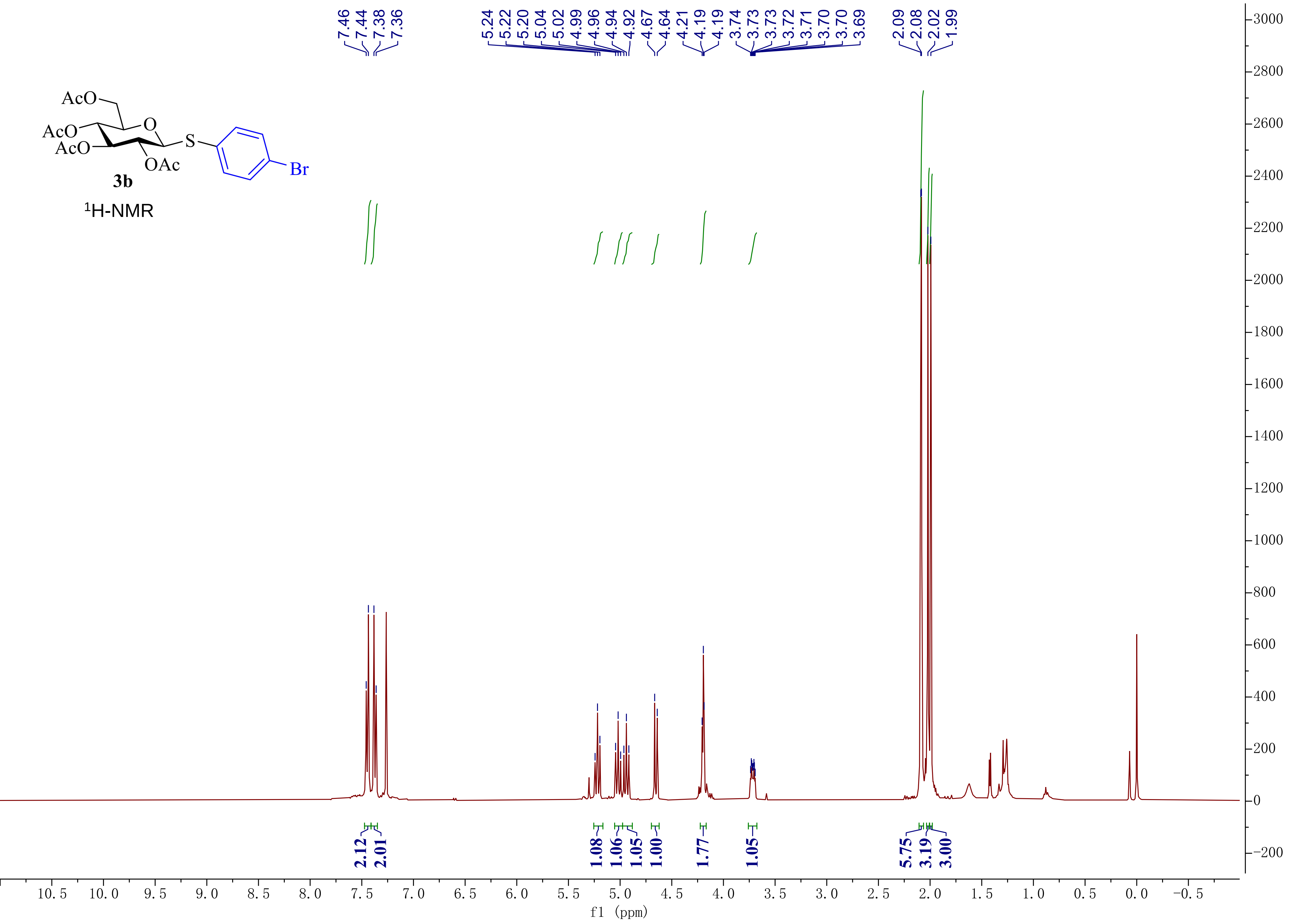

AcO
AcO
AcO
O
S
OAc
Br
3b
1H-NMR
7.46
7.44
7.38
7.36
5.24
5.22
5.20
5.04
5.02
4.99
4.96
4.94
4.92
4.67
4.64
4.21
4.19
4.19
3.74
3.73
3.73
3.72
3.71
3.70
3.70
3.69
2.09
2.08
2.02
1.99
2.12
2.01
1.08
1.06
1.05
1.00
1.77
1.05
5.75
3.19
3.00
f1 (ppm)

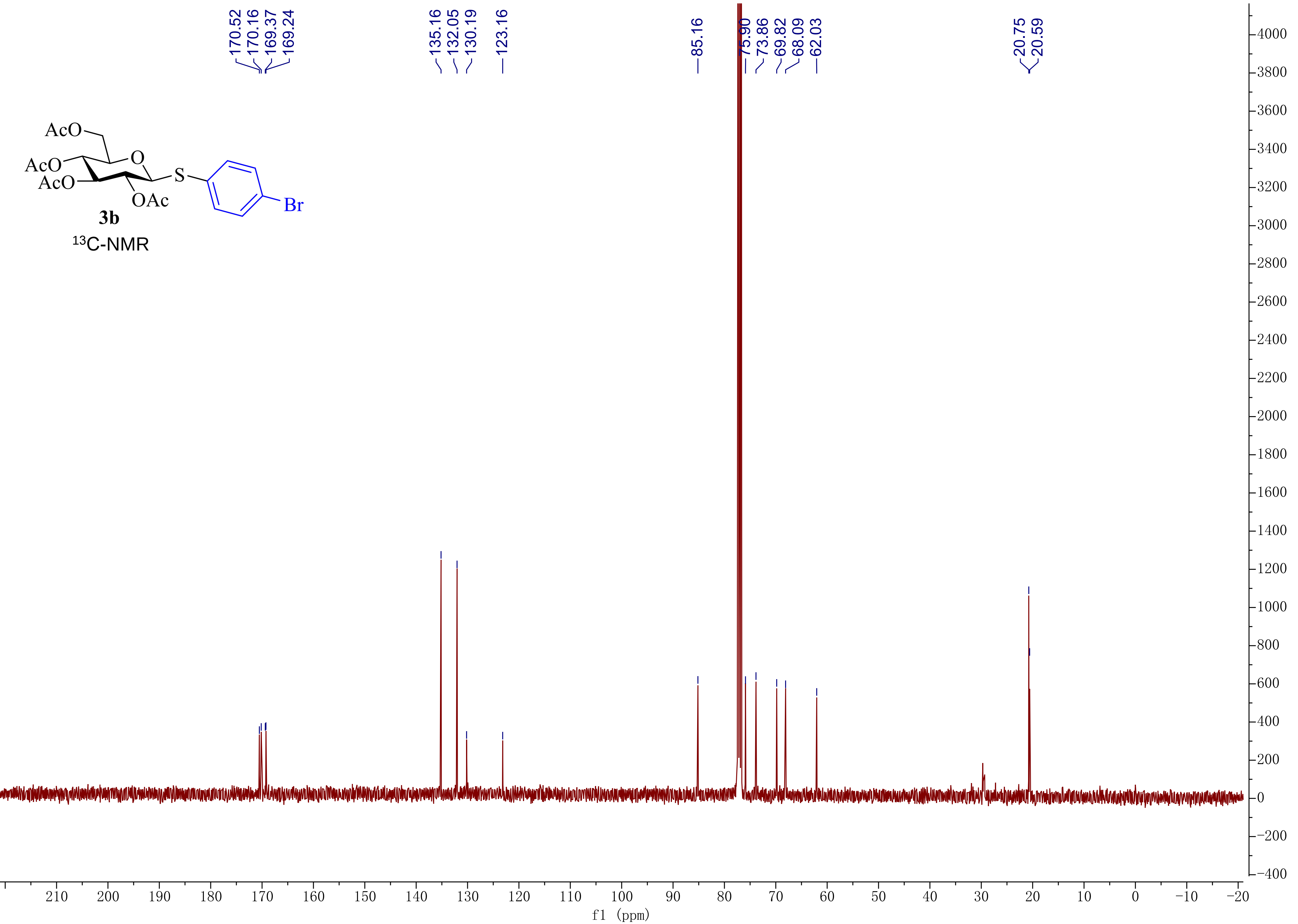
170.52
170.16
169.37
169.24
135.16
132.05
130.19
123.16
85.16
75.90
73.86
69.82
68.09
62.03
20.75
20.59
AcO
AcO
AcO
OAc
O
S
Br
3b
13C-NMR
f1 (ppm)

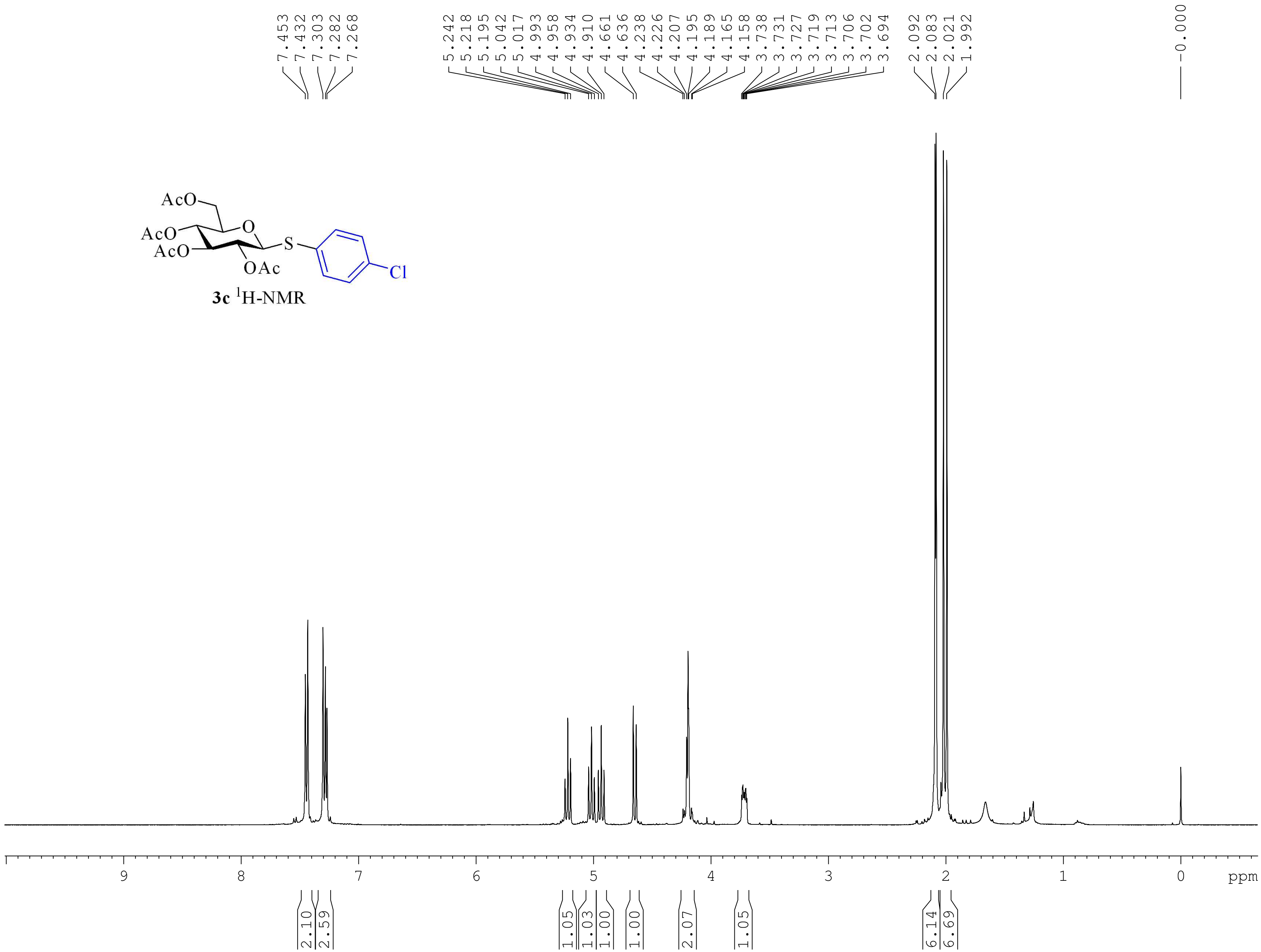

7.453
7.432
7.303
7.282
7.268
5.242
5.218
5.195
5.042
5.017
4.993
4.958
4.934
4.910
4.661
4.636
4.238
4.226
4.207
4.195
4.189
4.165
4.158
3.738
3.731
3.727
3.719
3.713
3.706
3.702
3.694
2.092
2.083
2.021
1.992
-0.000
AcO
AcO
AcO
O
S
OAc
Cl
9
8
7
6
5
4
3
2
1
0
ppm
2.10
2.59
1.05
1.03
1.00
1.00
2.07
1.05
6.14
6.69

3c ¹H-NMR

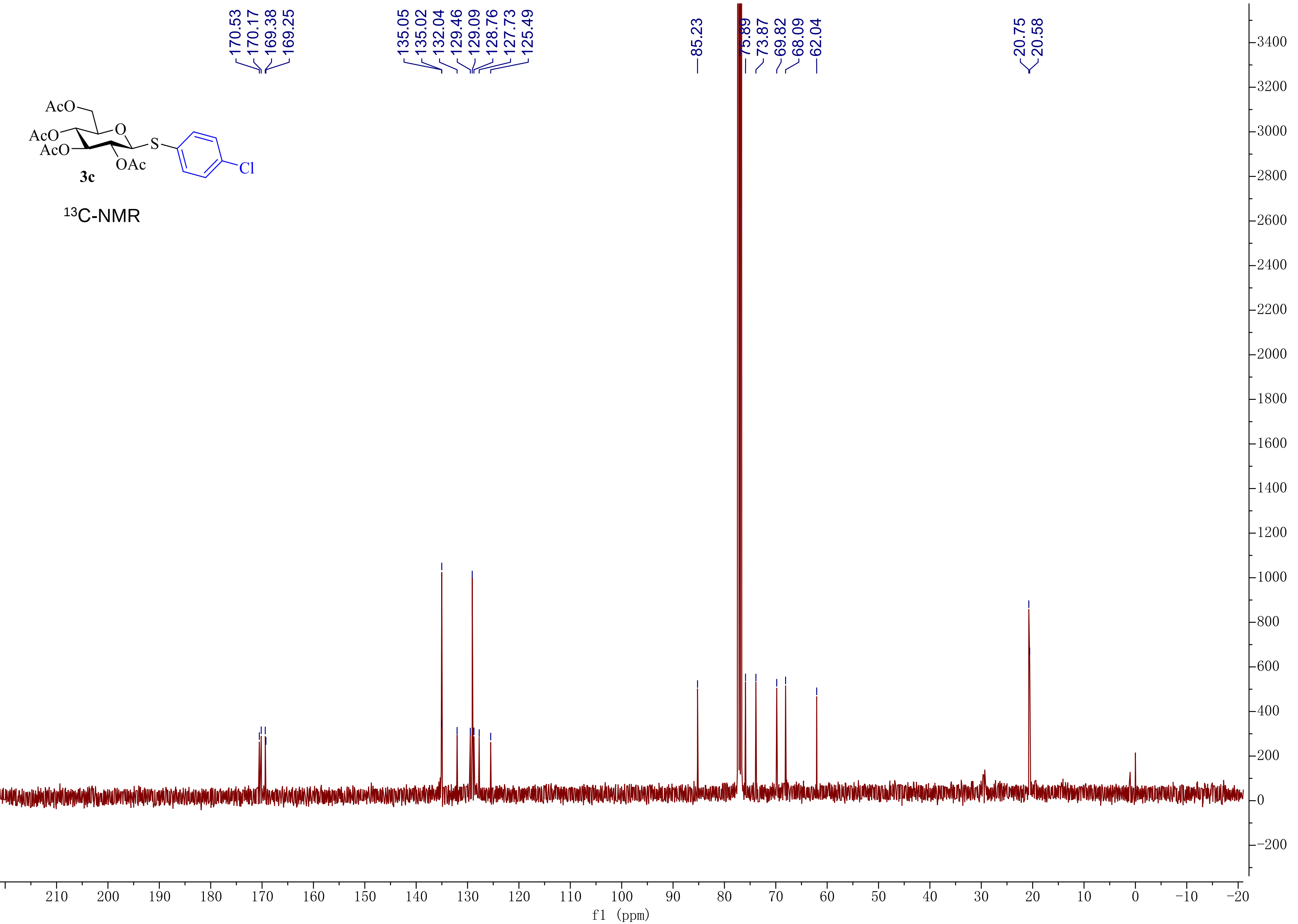

170.53
170.17
169.38
169.25
135.05
135.02
132.04
129.46
129.09
128.76
127.73
125.49
85.23
75.89
73.87
69.82
68.09
62.04
20.75
20.58
AcO
AcO
AcO
O
S
OAc
Cl
3c
13C-NMR
f1 (ppm)

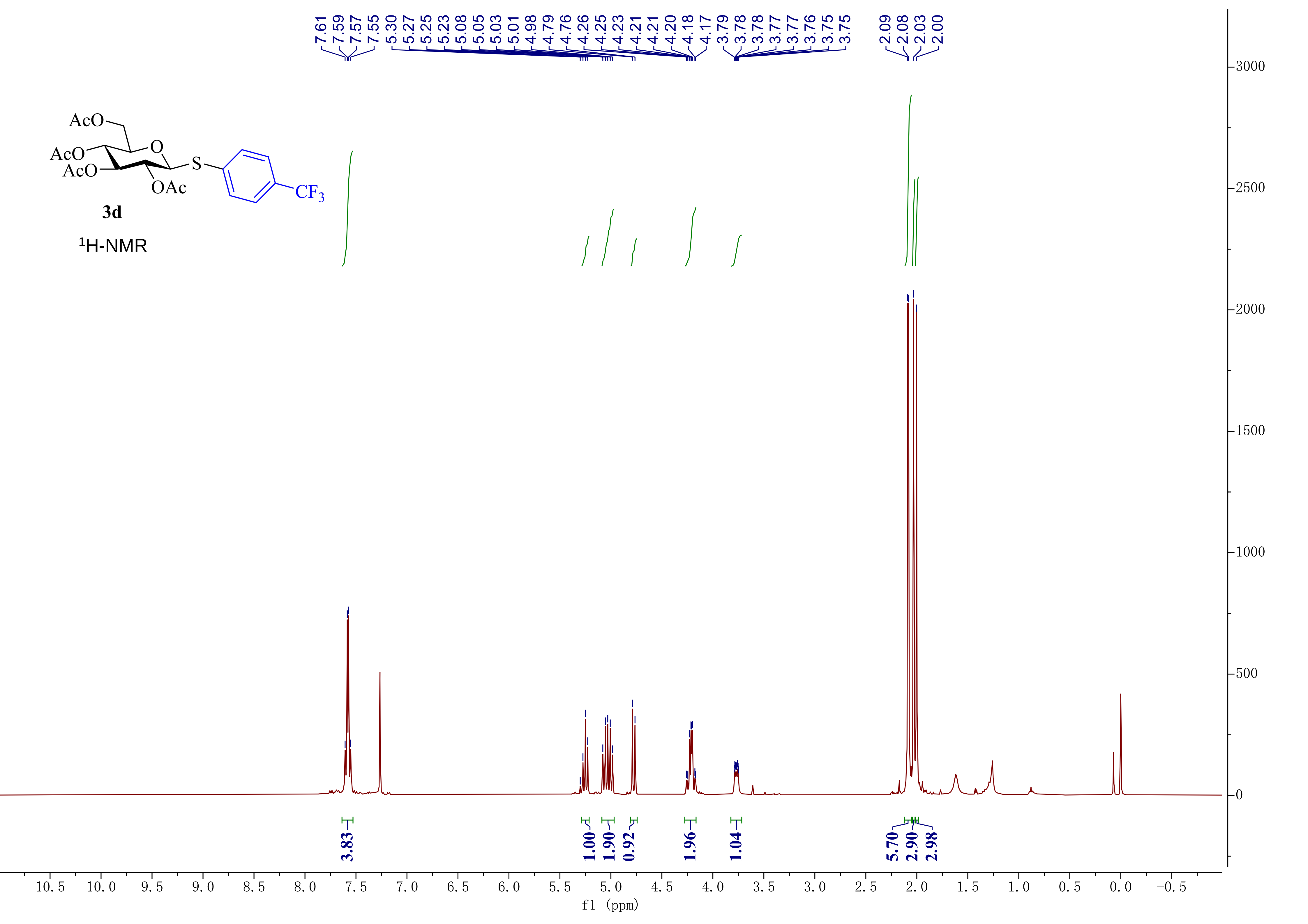

AcO
AcO
AcO
O
S
OAc
CF3
3d
1H-NMR
7.61 7.59 7.57 7.55 5.30 5.27 5.25 5.23 5.08 5.05 5.03 5.01 4.98 4.79 4.76 4.26 4.25 4.23 4.21 4.21 4.20 4.18 4.17 3.79 3.78 3.78 3.77 3.77 3.76 3.75 3.75 2.09 2.08 2.03 2.00
3.83
1.00
1.90
0.92
1.96
1.04
5.70
2.90
2.98
3000
2500
2000
1500
1000
500
0
10.5 10.0 9.5 9.0 8.5 8.0 7.5 7.0 6.5 6.0 5.5 5.0 4.5 4.0 3.5 3.0 2.5 2.0 1.5 1.0 0.5 0.0 -0.5
f1 (ppm)

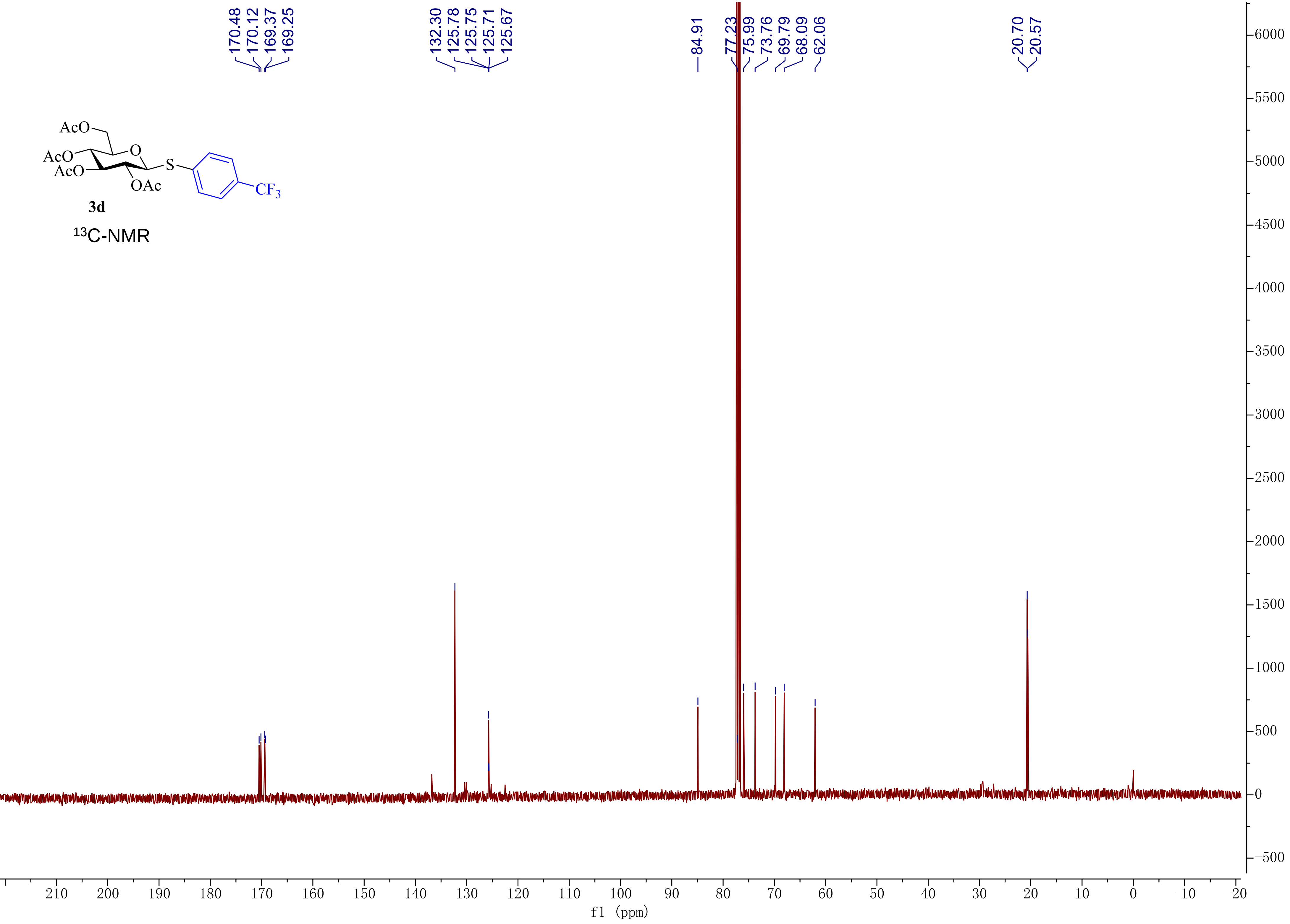
170.48
170.12
169.37
169.25
132.30
125.78
125.75
125.71
125.67
84.91
77.23
75.99
73.76
69.79
68.09
62.06
20.70
20.57
AcO
AcO
AcO
O
S
OAc
CF3
3d
13C-NMR
f1 (ppm)

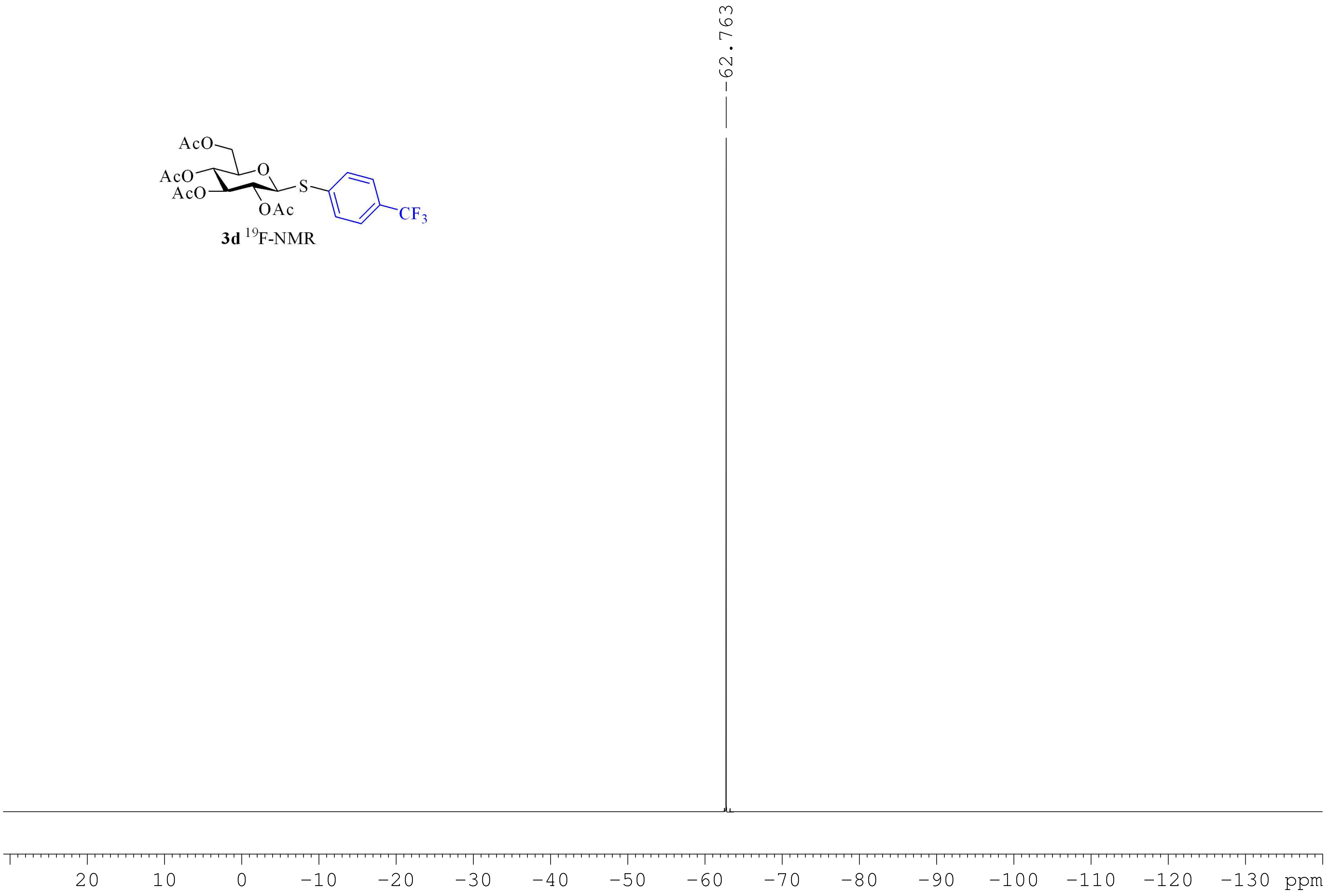
-62.763
AcO
AcO
AcO
O
S
OAc
CF3
3d 19F-NMR
20 10 0 -10 -20 -30 -40 -50 -60 -70 -80 -90 -100 -110 -120 -130 ppm

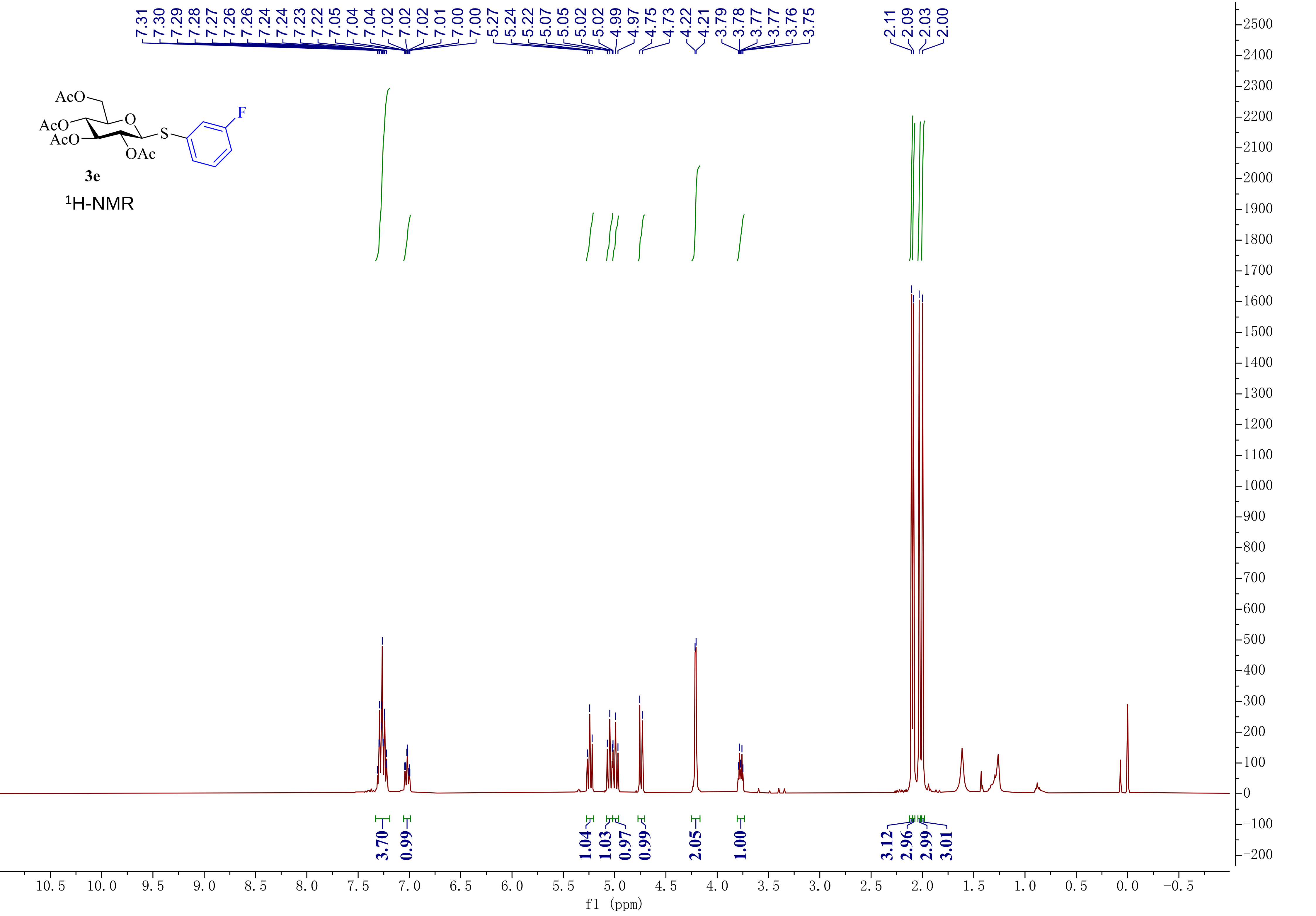

AcO
AcO
AcO
O
S
F
OAc
3e
¹H-NMR
7.31 7.30 7.29 7.28 7.27 7.26 7.26 7.24 7.24 7.23 7.22 7.05 7.04 7.04 7.02 7.02 7.02 7.01 7.00 7.00 5.27 5.24 5.22 5.07 5.05 5.02 5.02 4.99 4.97 4.75 4.73 4.22 4.21 3.79 3.78 3.77 3.77 3.76 3.75 2.11 2.09 2.03 2.00
3.70 0.99 1.04 1.03 0.97 0.99 2.05 1.00 3.12 2.96 2.99 3.01
f1 (ppm)

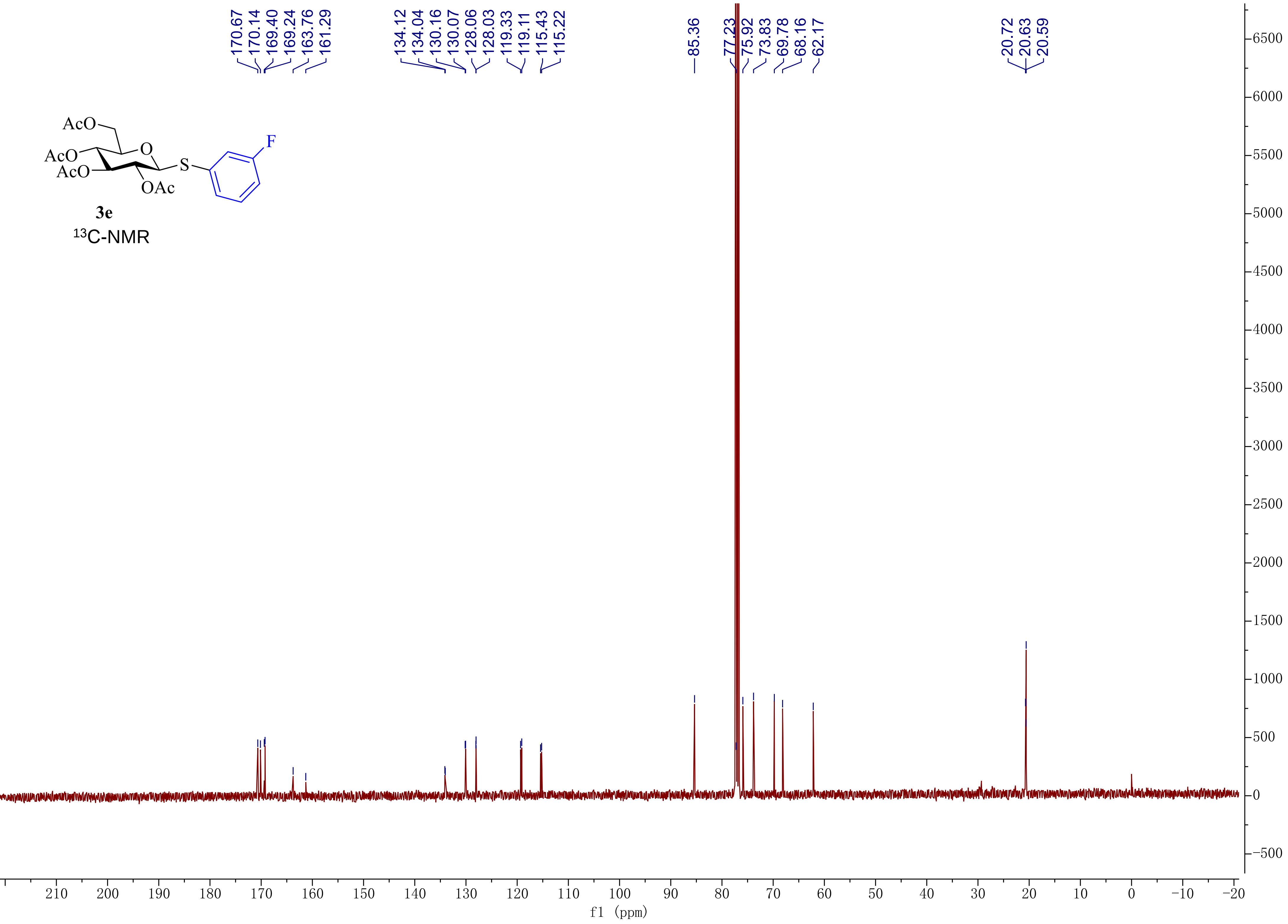

170.67
170.14
169.40
169.24
163.76
161.29
134.12
134.04
130.16
130.07
128.06
128.03
119.33
119.11
115.43
115.22
85.36
77.23
75.92
73.83
69.78
68.16
62.17
20.72
20.63
20.59
AcO
AcO
AcO
O
S
F
OAc
3e
13C-NMR
6500
6000
5500
5000
4500
4000
3500
3000
2500
2000
1500
1000
500
0
−500
210 200 190 180 170 160 150 140 130 120 110 100 90 80 70 60 50 40 30 20 10 0 −10 −20
f1 (ppm)

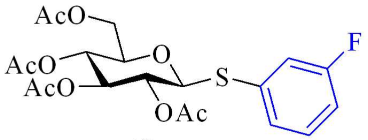

3e ^{19}F-NMR

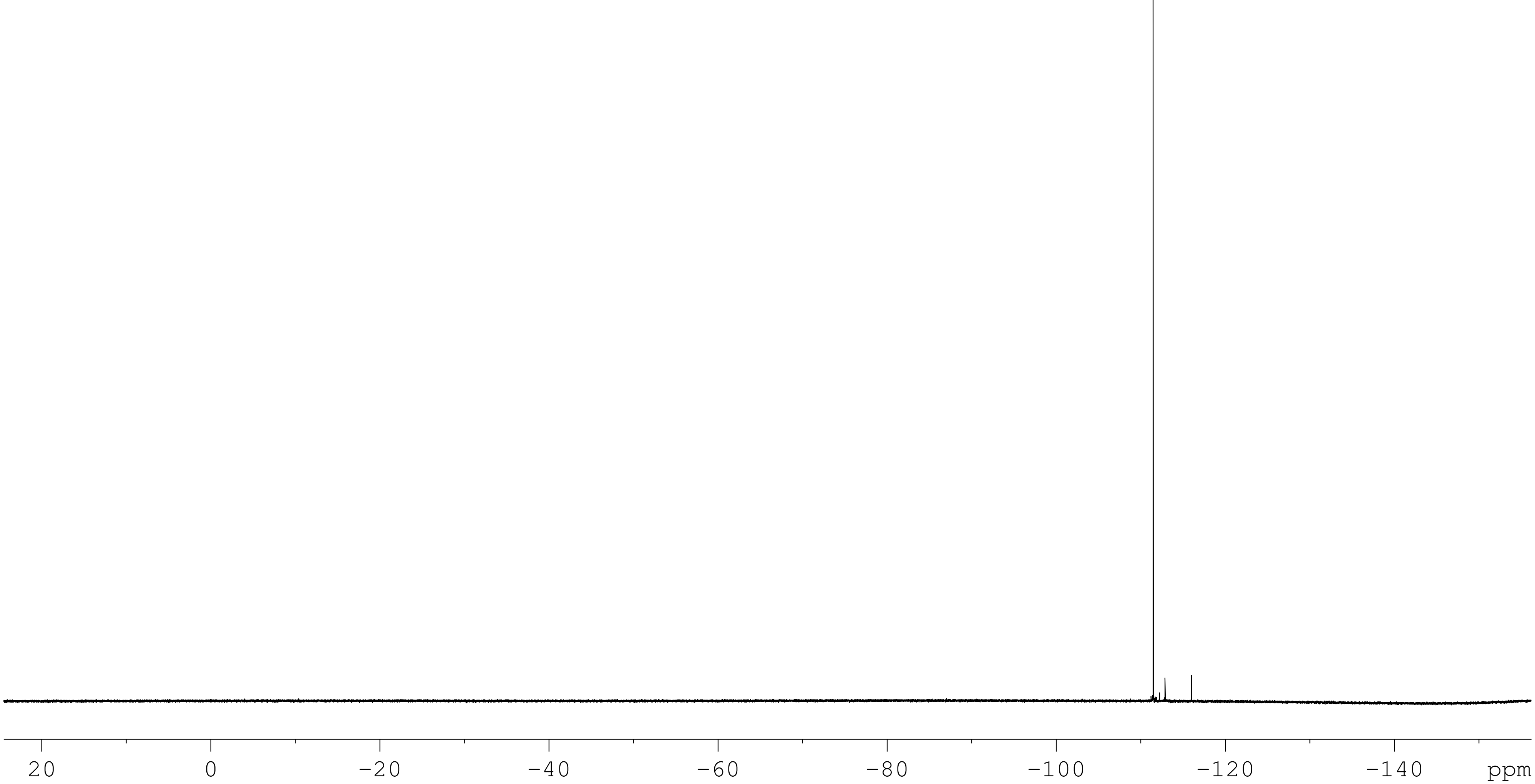

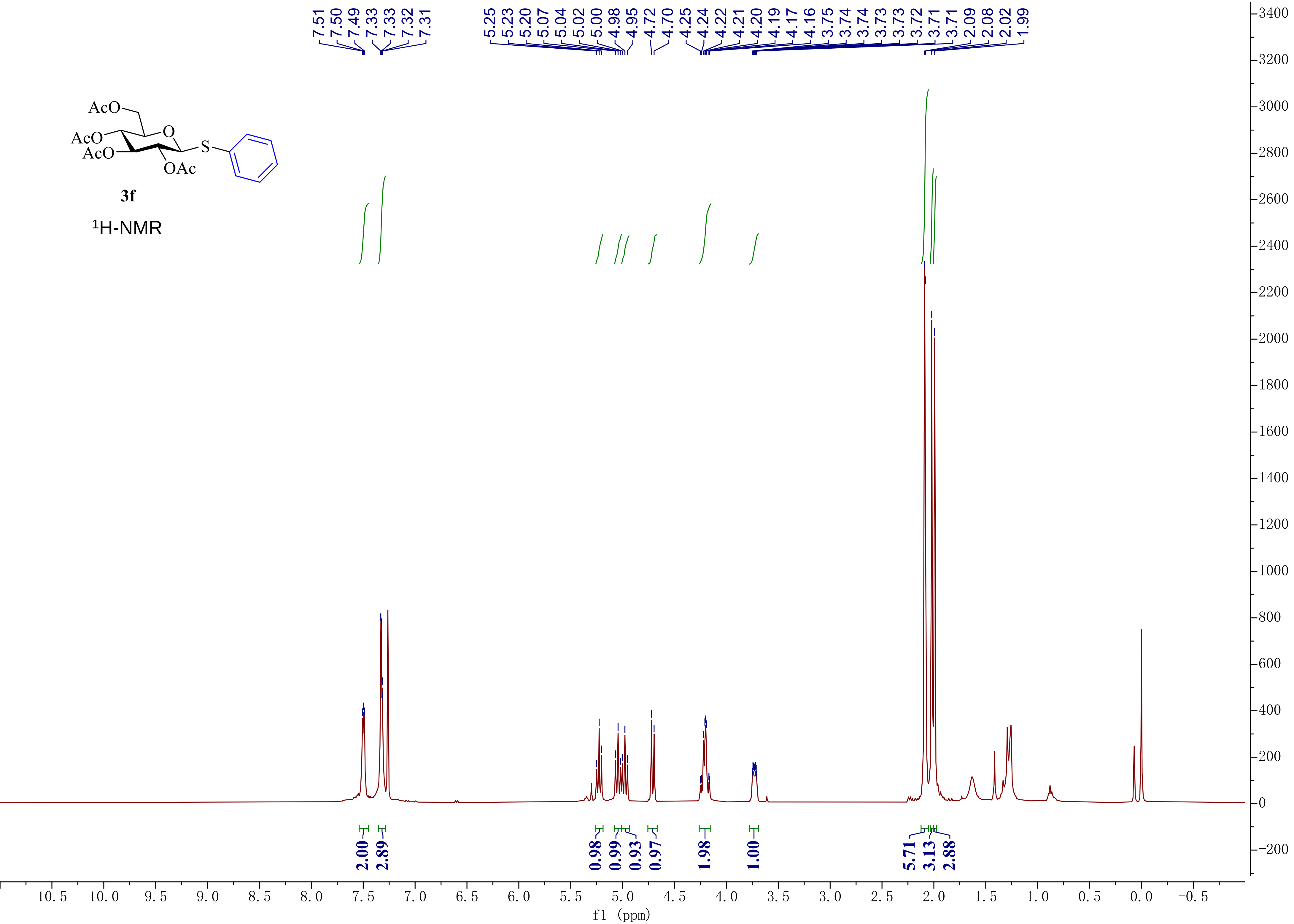

AcO
AcO
AcO
O
S
OAc
3f
1H-NMR
7.51 7.50 7.49 7.33 7.33 7.32 7.31
5.25 5.23 5.20 5.07 5.04 5.02 5.00 4.98 4.95 4.72 4.70
4.25 4.24 4.22 4.21 4.20 4.19 4.17 4.16 3.75 3.74 3.74 3.73 3.73 3.72 3.71 3.71 2.09 2.08 2.02 1.99
2.00 2.89 0.98 0.99 0.93 0.97 1.98 1.00 5.71 3.13 2.88
f1 (ppm)

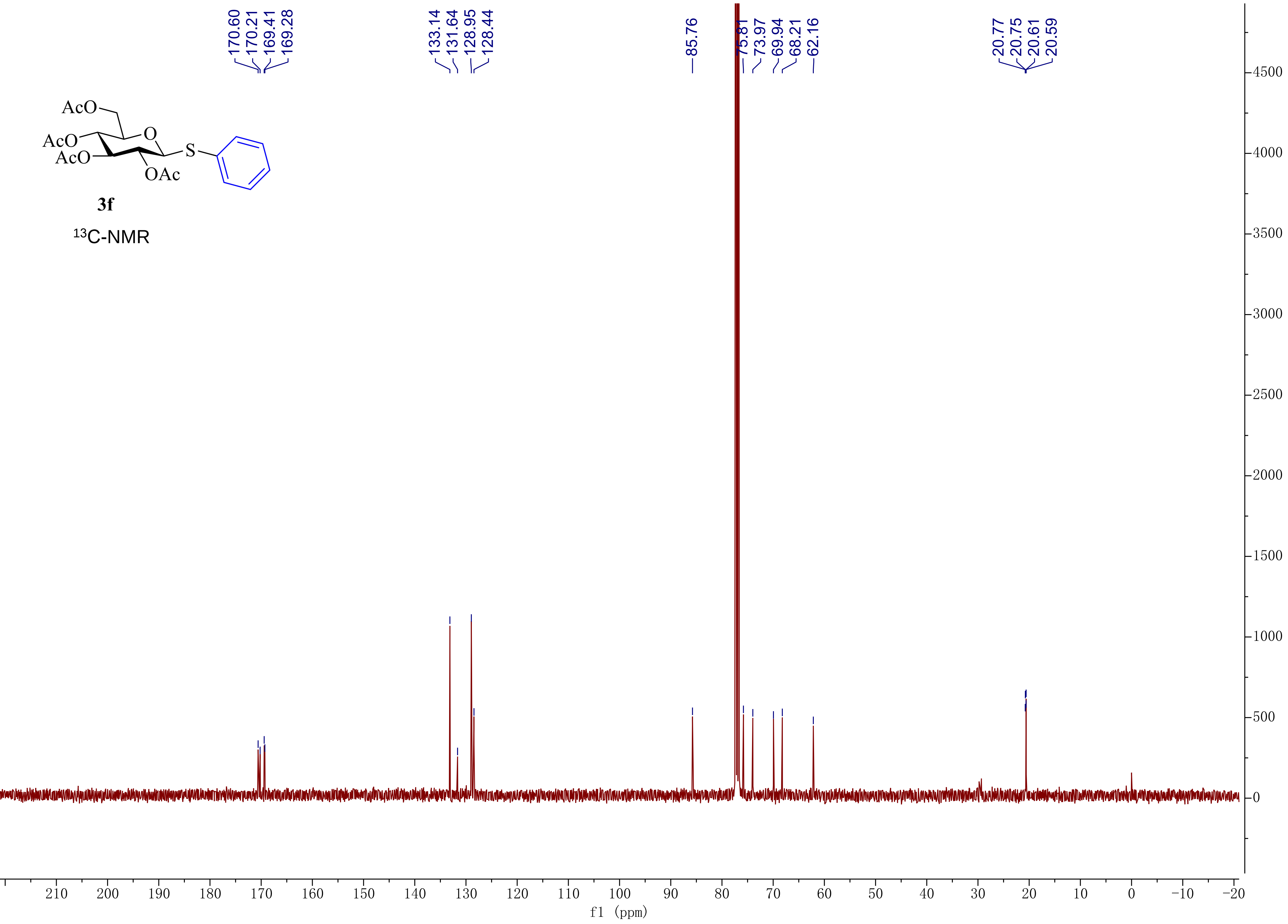
AcO
AcO
AcO
O
S
OAc
3f
13C-NMR
170.60
170.21
169.41
169.28
133.14
131.64
128.95
128.44
85.76
75.81
73.97
69.94
68.21
62.16
20.77
20.75
20.61
20.59
f1 (ppm)

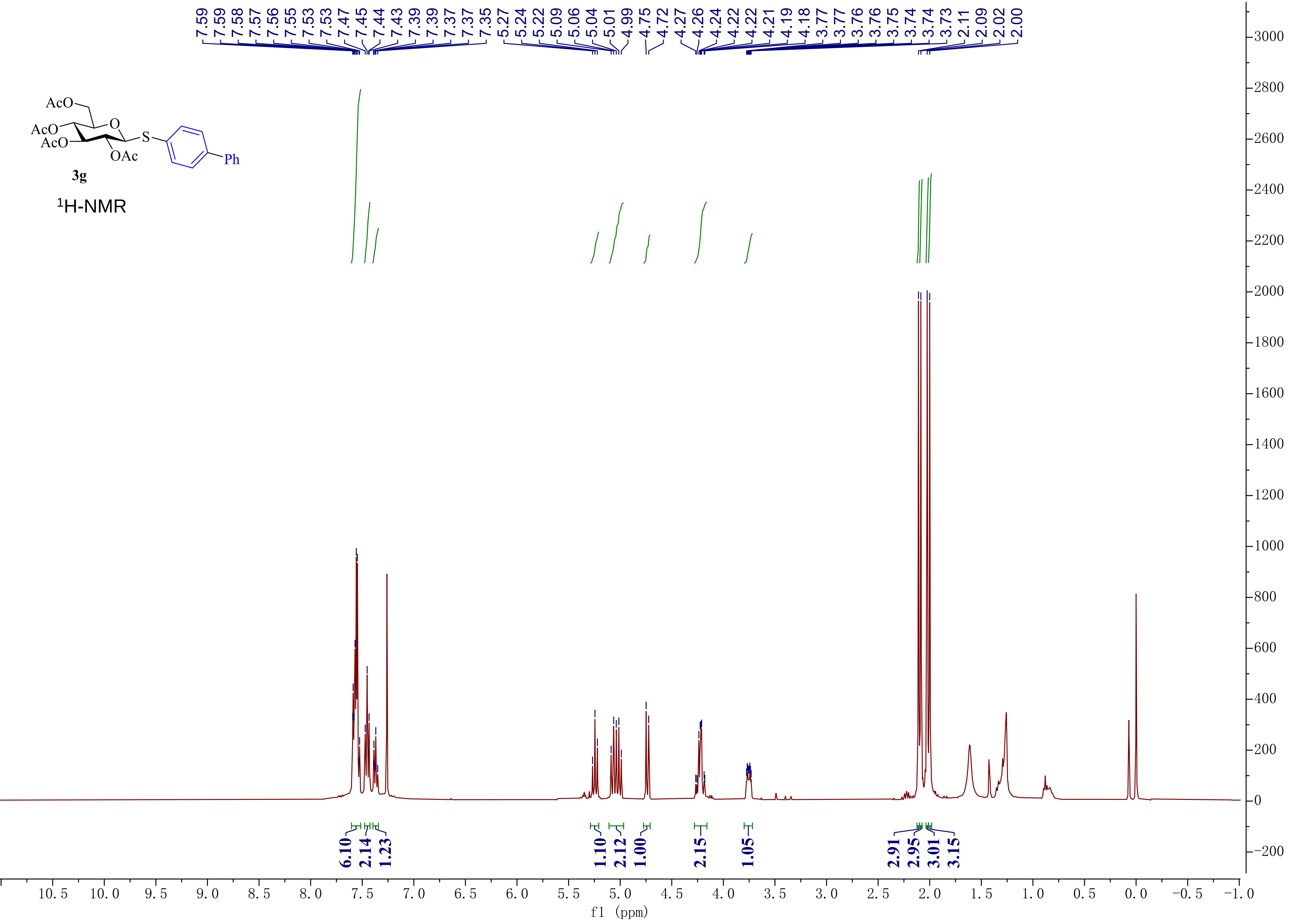

7.59 7.59 7.58 7.57 7.56 7.55 7.53 7.53 7.47 7.45 7.44 7.43 7.39 7.39 7.37 7.37 7.35 5.27 5.24 5.22 5.09 5.06 5.04 5.01 4.99 4.75 4.72 4.27 4.26 4.24 4.22 4.22 4.21 4.19 4.18 3.77 3.77 3.76 3.76 3.75 3.74 3.74 3.73 2.11 2.09 2.02 2.00
AcO
AcO
AcO
O
S
OAc
Ph
3g
1H-NMR
6.10 2.14 1.23 1.10 2.12 1.00 2.15 1.05 2.91 2.95 3.01 3.15
3000 2800 2600 2400 2200 2000 1800 1600 1400 1200 1000 800 600 400 200 0 -200
10.5 10.0 9.5 9.0 8.5 8.0 7.5 7.0 6.5 6.0 5.5 5.0 4.5 4.0 3.5 3.0 2.5 2.0 1.5 1.0 0.5 0.0 -0.5 -1.0
f1 (ppm)

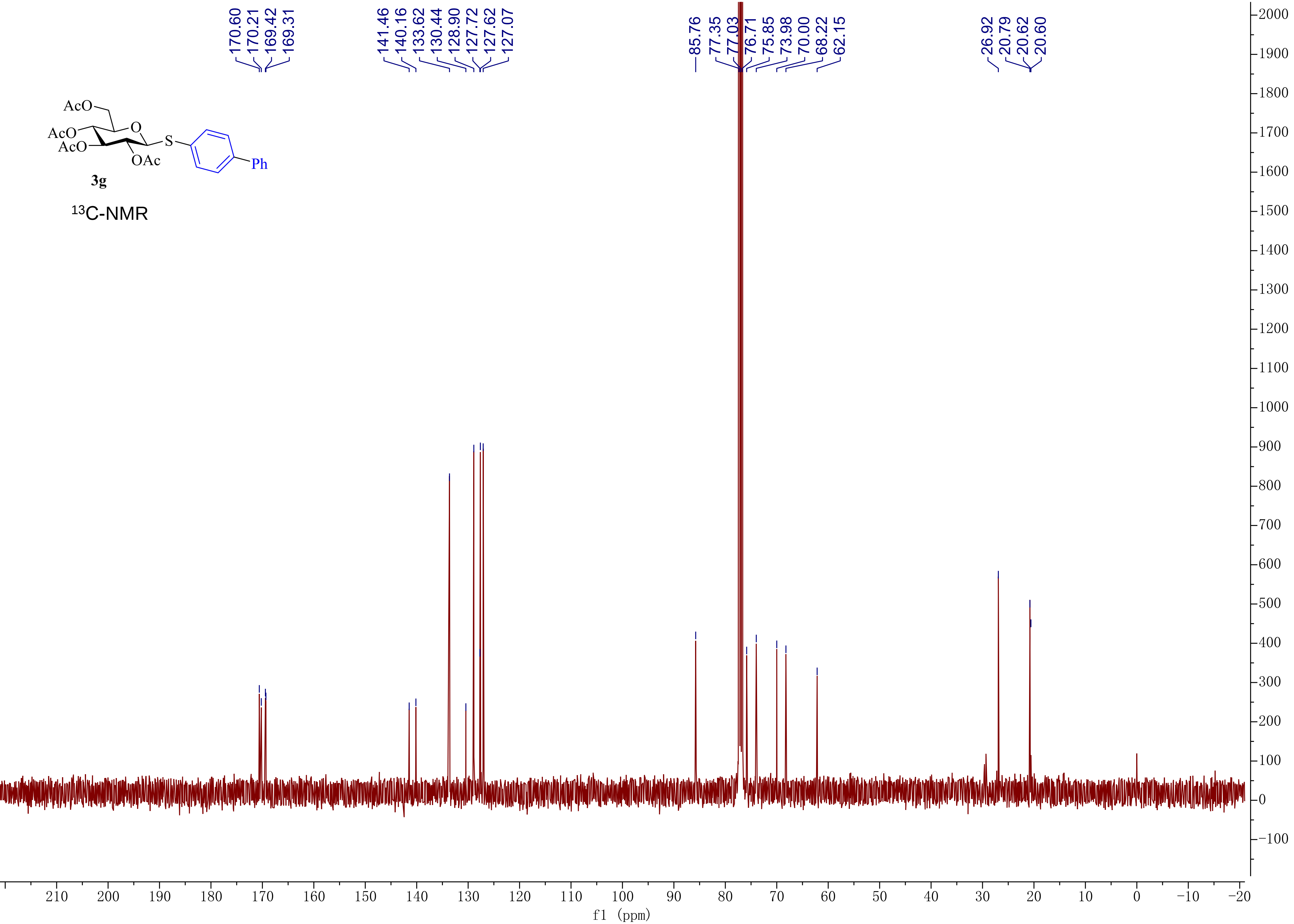
AcO
AcO
AcO
O
S
OAc
Ph
170.60
170.21
169.42
169.31
141.46
140.16
133.62
130.44
128.90
127.72
127.62
127.07
85.76
77.35
77.03
76.71
75.85
73.98
70.00
68.22
62.15
26.92
20.79
20.62
20.60
210 200 190 180 170 160 150 140 130 120 110 100 90 80 70 60 50 40 30 20 10 0 −10 −20
f1 (ppm)
2000 1900 1800 1700 1600 1500 1400 1300 1200 1100 1000 900 800 700 600 500 400 300 200 100 0 −100

3g

^{13}C-NMR

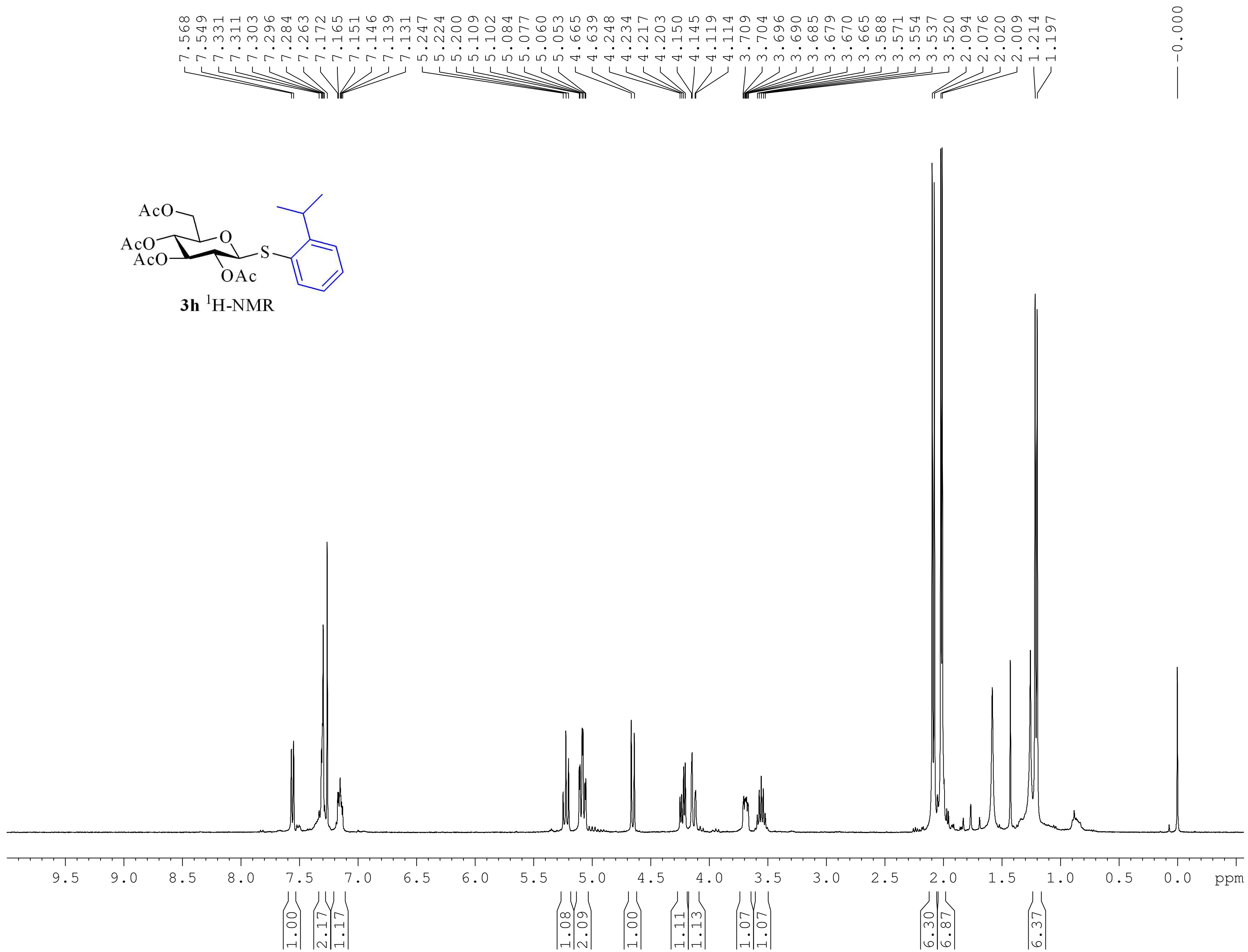

7.568
7.549
7.331
7.311
7.303
7.296
7.284
7.263
7.172
7.165
7.151
7.146
7.139
7.131
5.247
5.224
5.200
5.109
5.102
5.084
5.077
5.060
5.053
4.665
4.639
4.248
4.234
4.217
4.203
4.150
4.145
4.119
4.114
3.709
3.704
3.696
3.690
3.685
3.679
3.670
3.665
3.588
3.571
3.554
3.537
3.520
2.094
2.076
2.020
2.009
1.214
1.197
-0.000
AcO
AcO
AcO
O
S
OAc
3h ^{1}H-NMR
9.5 9.0 8.5 8.0 7.5 7.0 6.5 6.0 5.5 5.0 4.5 4.0 3.5 3.0 2.5 2.0 1.5 1.0 0.5 0.0 ppm
1.00
2.17
1.17
1.08
2.09
1.00
1.11
1.13
1.07
1.07
6.30
6.87
6.37

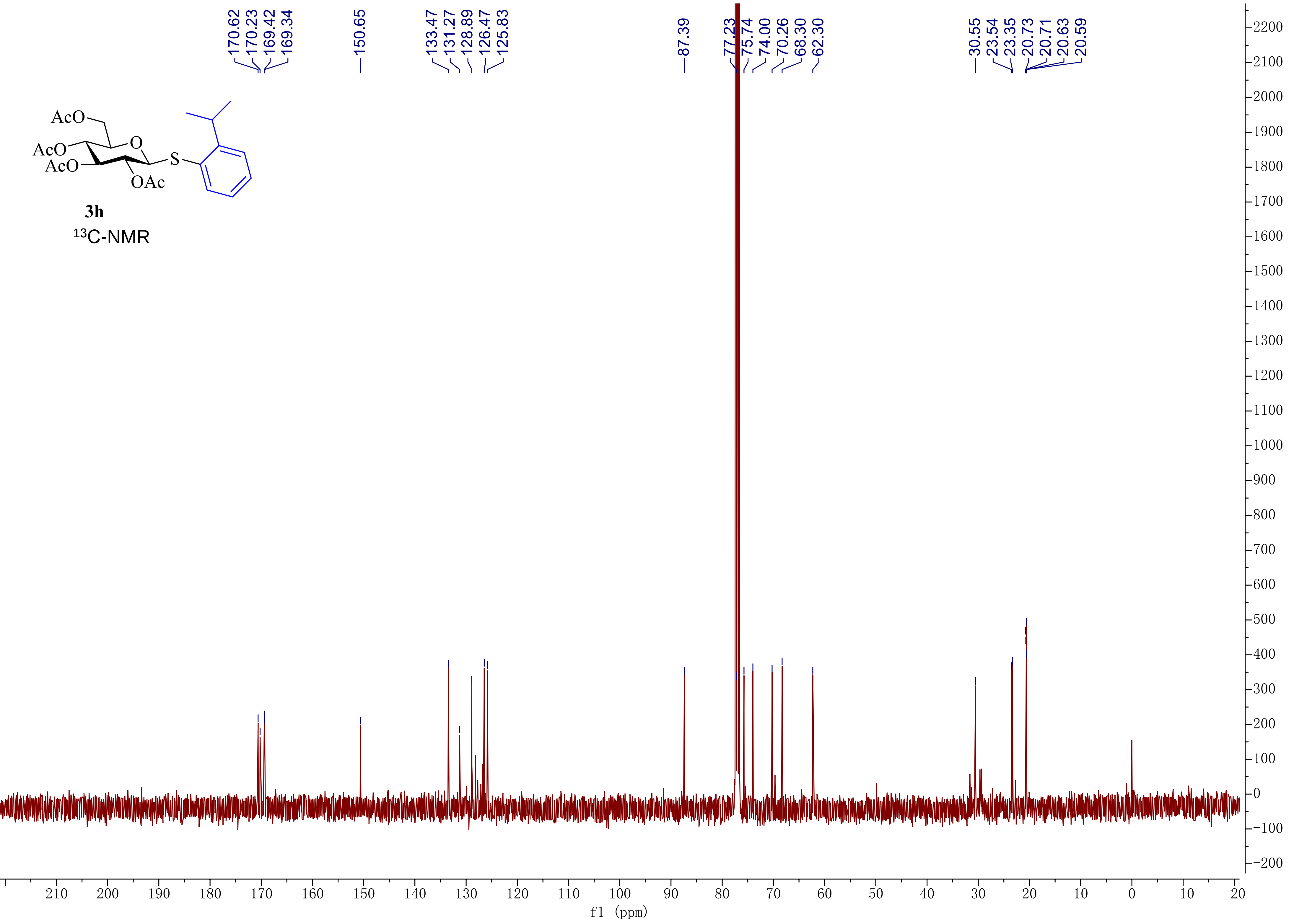

170.62
170.23
169.42
169.34
150.65
133.47
131.27
128.89
126.47
125.83
87.39
77.23
75.74
74.00
70.26
68.30
62.30
30.55
23.54
23.35
20.73
20.71
20.63
20.59
AcO
AcO
AcO
O
S
OAc
3h
13C-NMR
f1 (ppm)

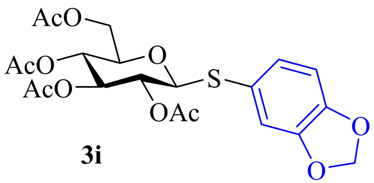
AcO
AcO
AcO
O
S
OAc
O
O

3i

^{1}H-NMR

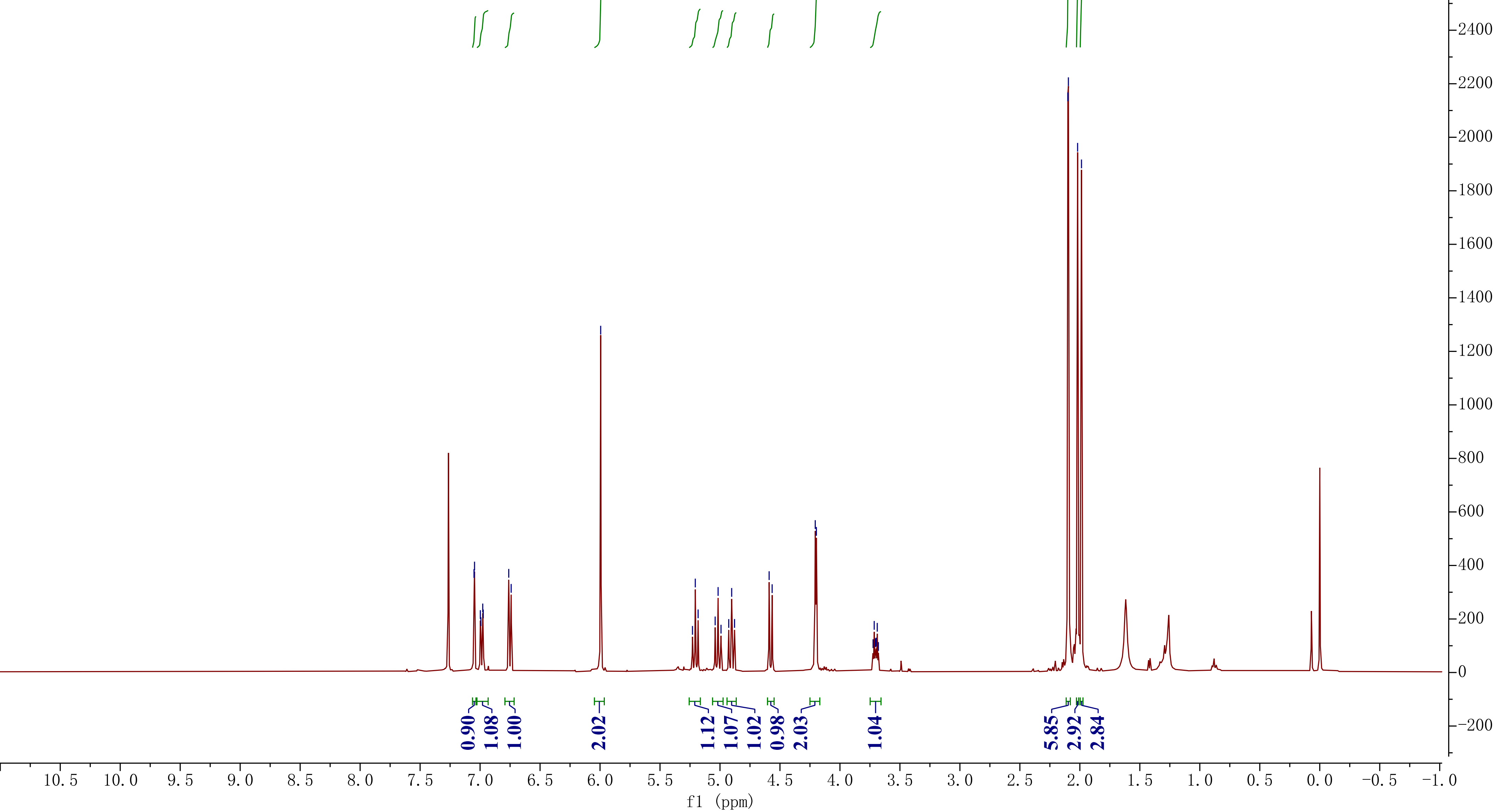
7.05
7.05
7.00
6.99
6.98
6.97
6.76
6.74
5.99
5.23
5.21
5.18
5.04
5.02
4.99
4.93
4.90
4.88
4.59
4.57
4.21
4.20
3.72
3.71
3.70
3.70
3.69
3.68
2.10
2.10
2.02
1.99
0.90
1.08
1.00
2.02
1.12
1.07
1.02
0.98
2.03
1.04
5.85
2.92
2.84
f1 (ppm)

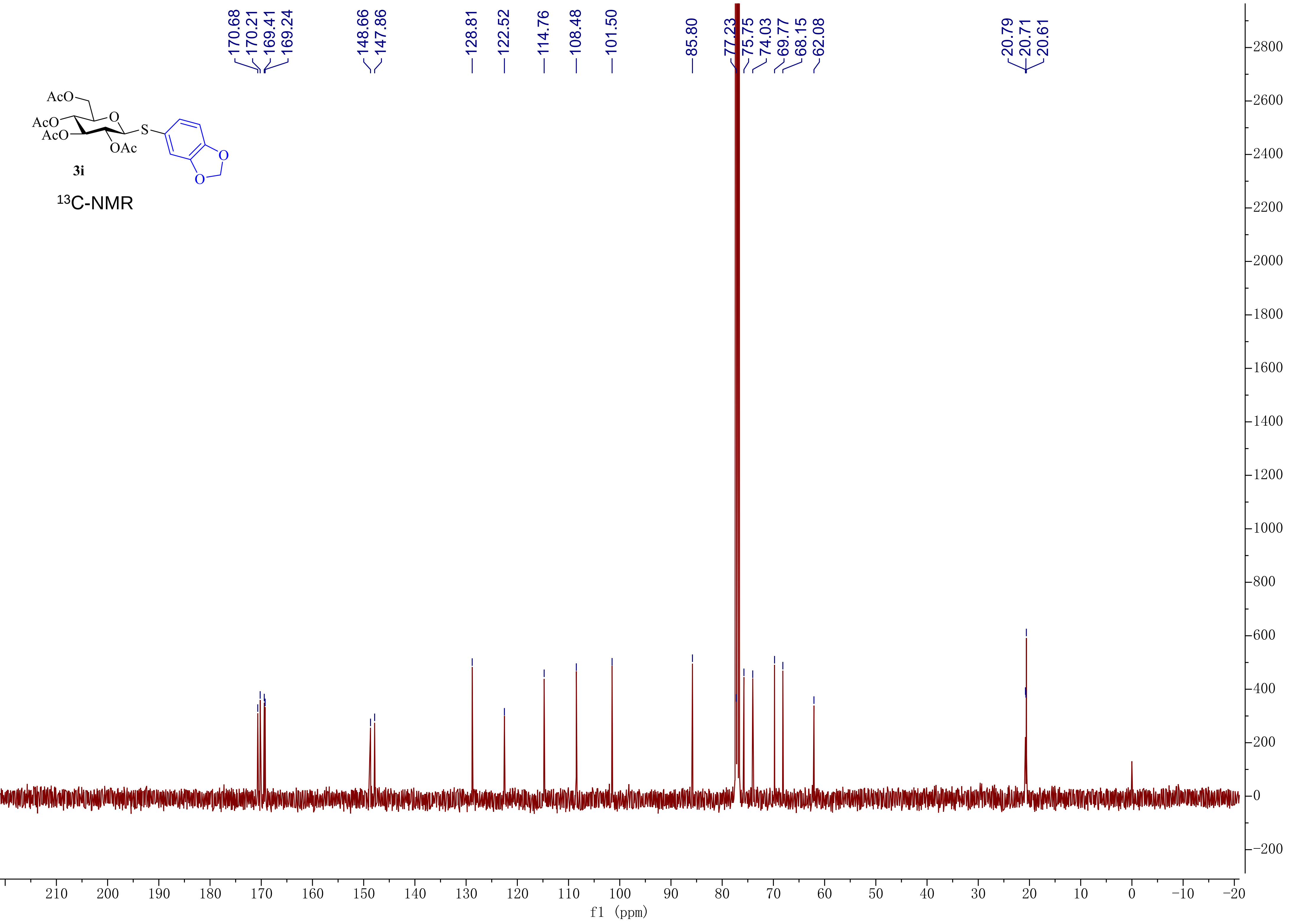

170.68
170.21
169.41
169.24
148.66
147.86
128.81
122.52
114.76
108.48
101.50
85.80
77.23
75.75
74.03
69.77
68.15
62.08
20.79
20.71
20.61
AcO
AcO
AcO
OAc
O
S
O
O
3i
13C-NMR
f1 (ppm)

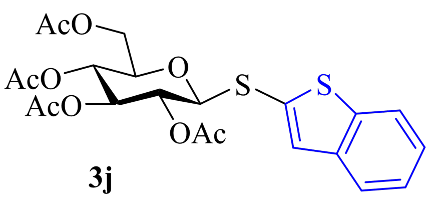
AcO
AcO
AcO
O
S
S
OAc

3j

^{1}H-NMR

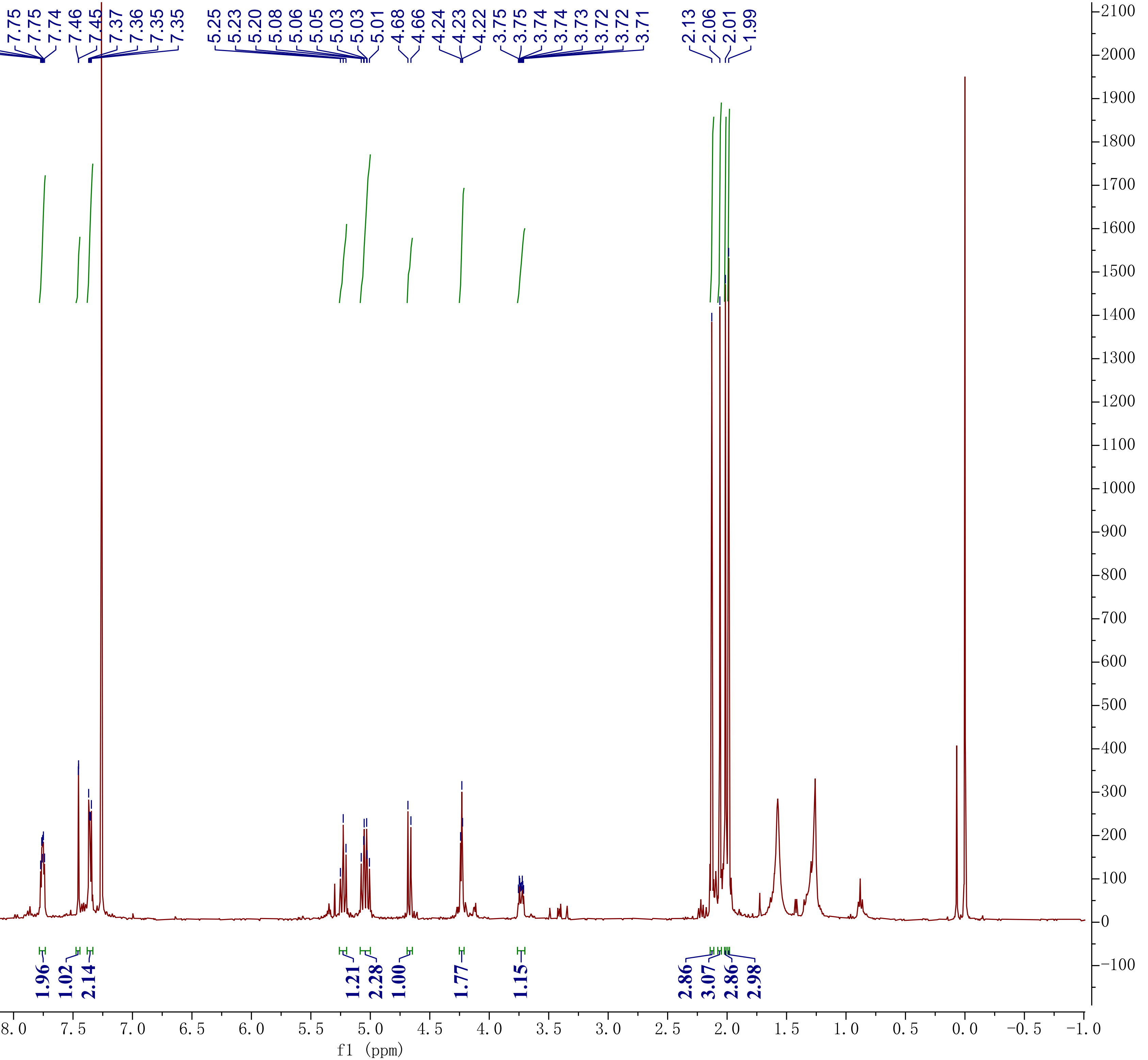
7.77 7.76 7.76 7.75 7.75 7.74 7.46 7.45 7.37 7.36 7.35 7.35
5.25 5.23 5.20 5.08 5.06 5.05 5.03 5.03 5.01 4.68 4.66 4.24 4.23 4.22 3.75 3.75 3.74 3.74 3.73 3.72 3.72 3.71
2.13 2.06 2.01 1.99
1.96 1.02 2.14 1.21 2.28 1.00 1.77 1.15 2.86 3.07 2.86 2.98
f1 (ppm)

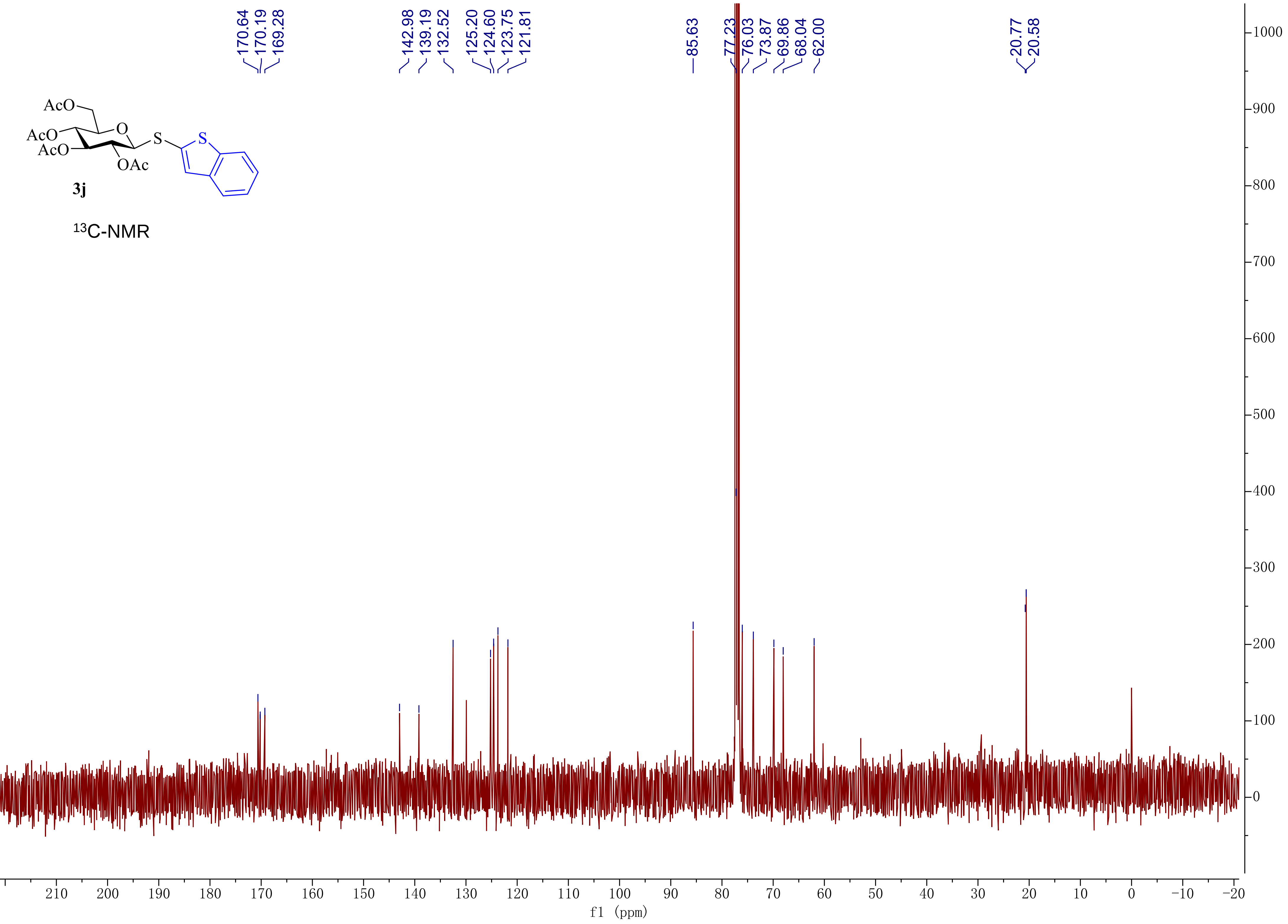
AcO
AcO
AcO
O
S
S
OAc
3j
13C-NMR
170.64
170.19
169.28
142.98
139.19
132.52
125.20
124.60
123.75
121.81
85.63
77.23
76.03
73.87
69.86
68.04
62.00
20.77
20.58
f1 (ppm)

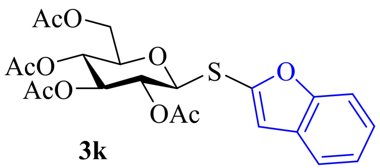
AcO
AcO
AcO
O
OAc
S
O

3k

¹H-NMR

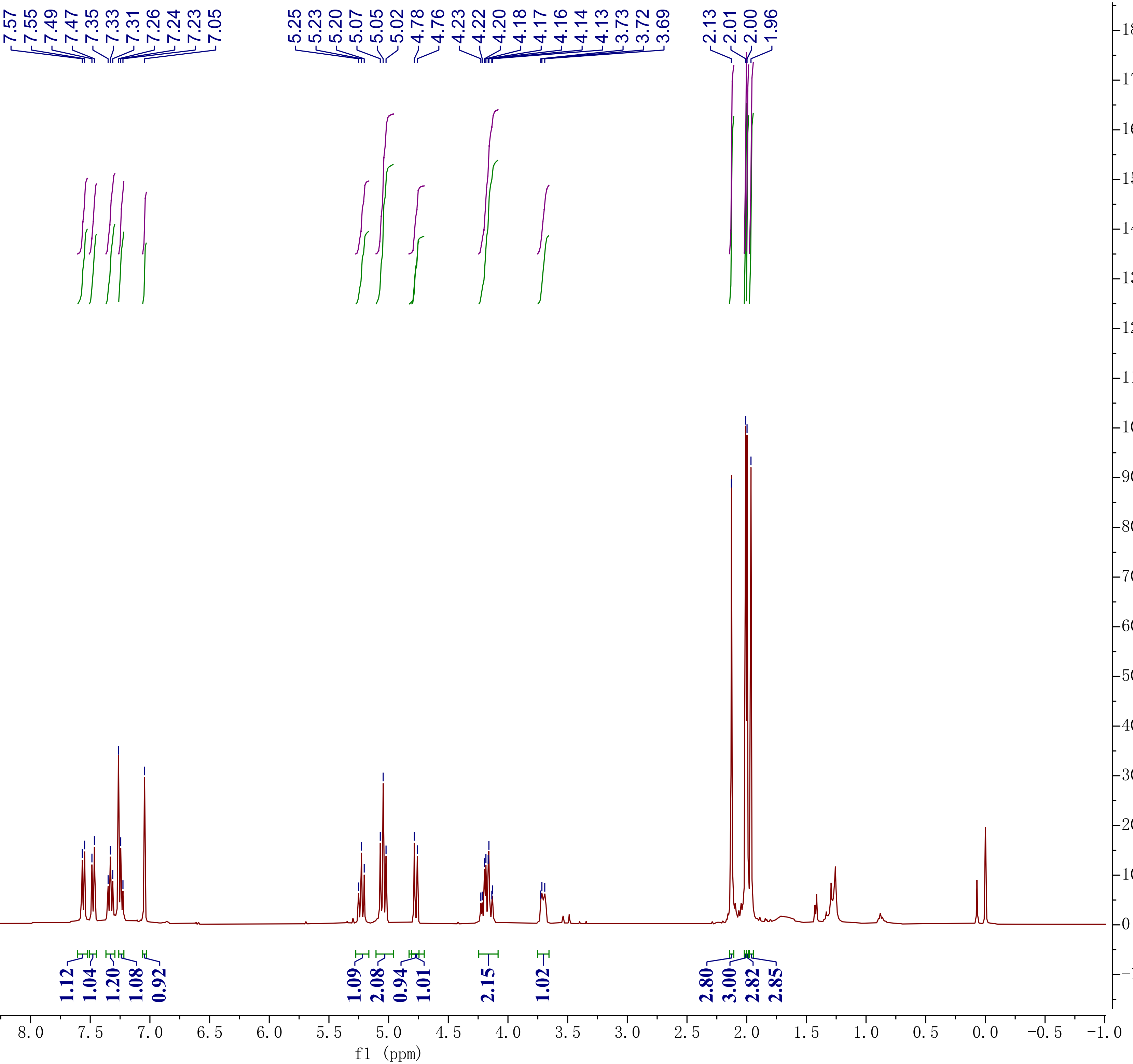
7.57 7.55 7.49 7.47 7.35 7.33 7.31 7.26 7.24 7.23 7.05
5.25 5.23 5.20 5.07 5.05 5.02 4.78 4.76 4.23 4.22 4.20 4.18 4.17 4.16 4.14 4.13 3.73 3.72 3.69
2.13 2.01 2.00 1.96
1.12 1.04 1.20 1.08 0.92
1.09 2.08 0.94 1.01
2.15
1.02
2.80 3.00 2.82 2.85
10.5 10.0 9.5 9.0 8.5 8.0 7.5 7.0 6.5 6.0 5.5 5.0 4.5 4.0 3.5 3.0 2.5 2.0 1.5 1.0 0.5 0.0 -0.5 -1.0
f1 (ppm)
1800 1700 1600 1500 1400 1300 1200 1100 1000 900 800 700 600 500 400 300 200 100 0 -100

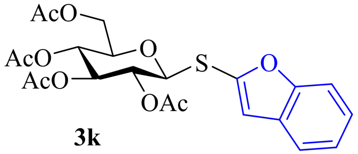

AcO
AcO
AcO
O
S
O
OAc

3k

^{13}C-NMR

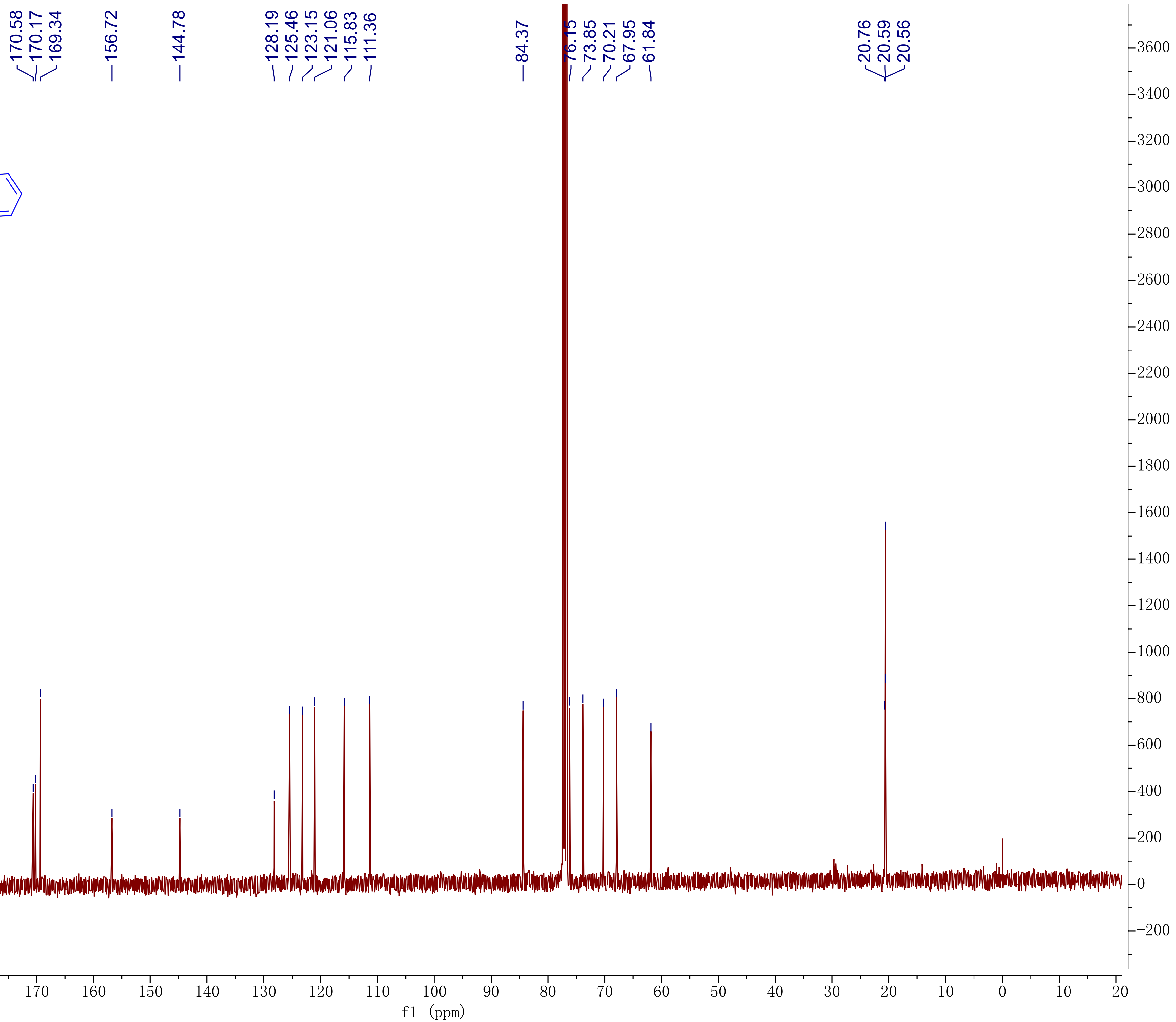

170.58
170.17
169.34
156.72
144.78
128.19
125.46
123.15
121.06
115.83
111.36
84.37
76.15
73.85
70.21
67.95
61.84
20.76
20.59
20.56
f1 (ppm)

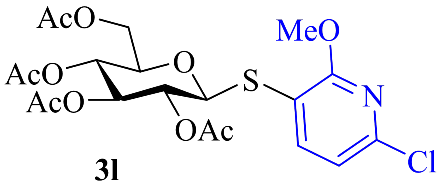

AcO
AcO
AcO
O
OAc
S
MeO
N
Cl

3l

^{1}H-NMR

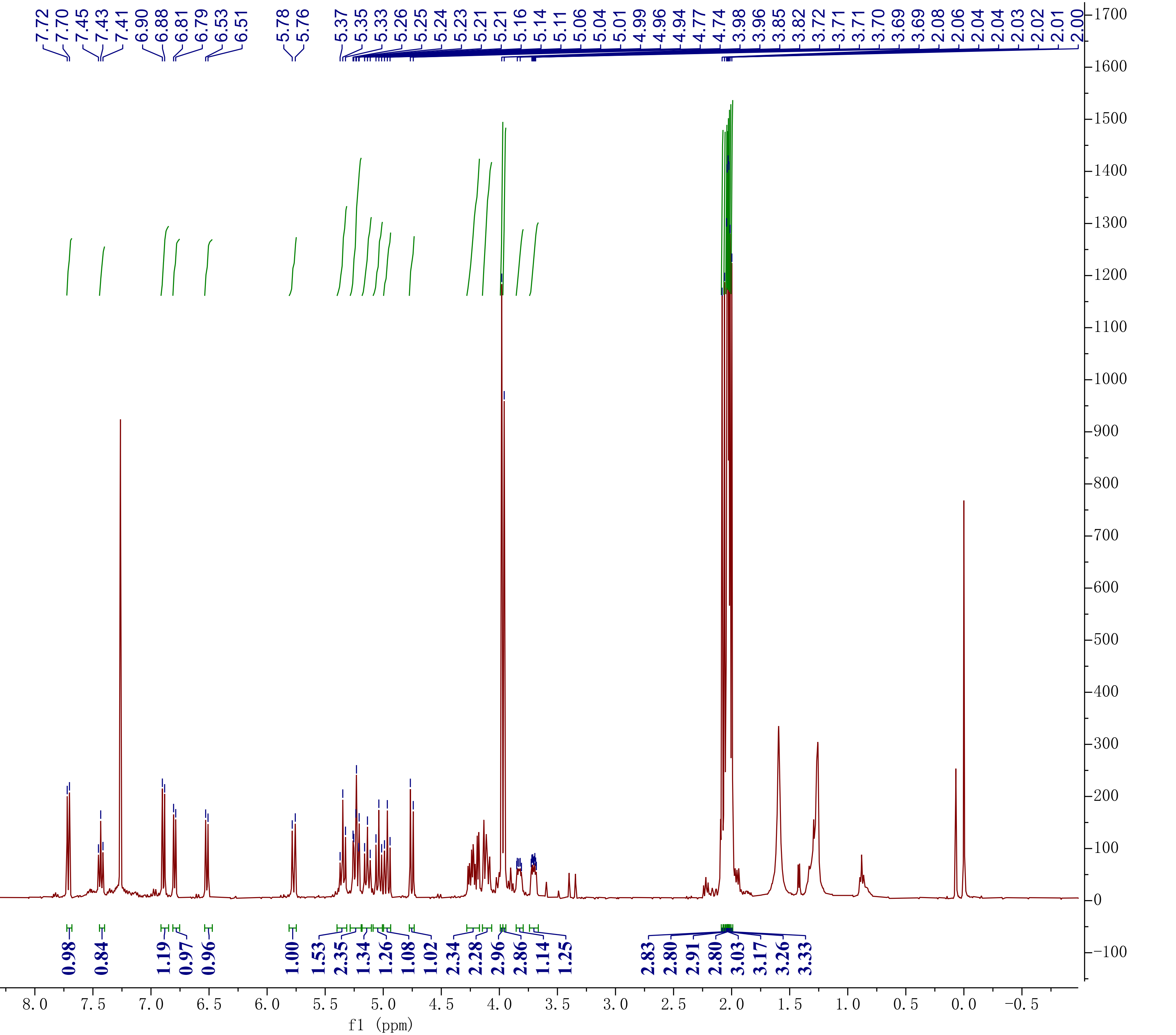

7.72 7.70 7.45 7.43 7.41 6.90 6.88 6.81 6.79 6.53 6.51 5.78 5.76 5.37 5.35 5.33 5.26 5.25 5.24 5.23 5.21 5.21 5.16 5.14 5.11 5.06 5.04 5.01 4.99 4.96 4.94 4.77 4.74 3.98 3.96 3.85 3.82 3.72 3.71 3.71 3.70 3.69 3.69 2.08 2.06 2.04 2.04 2.03 2.02 2.01 2.00
0.98 0.84 1.19 0.97 0.96 1.00 1.53 2.35 1.34 1.26 1.08 1.02 2.34 2.28 2.96 2.86 1.14 1.25 2.83 2.80 2.91 2.80 3.03 3.17 3.26 3.33
1700 1600 1500 1400 1300 1200 1100 1000 900 800 700 600 500 400 300 200 100 0 −100
10.5 10.0 9.5 9.0 8.5 8.0 7.5 7.0 6.5 6.0 5.5 5.0 4.5 4.0 3.5 3.0 2.5 2.0 1.5 1.0 0.5 0.0 −0.5
f1 (ppm)

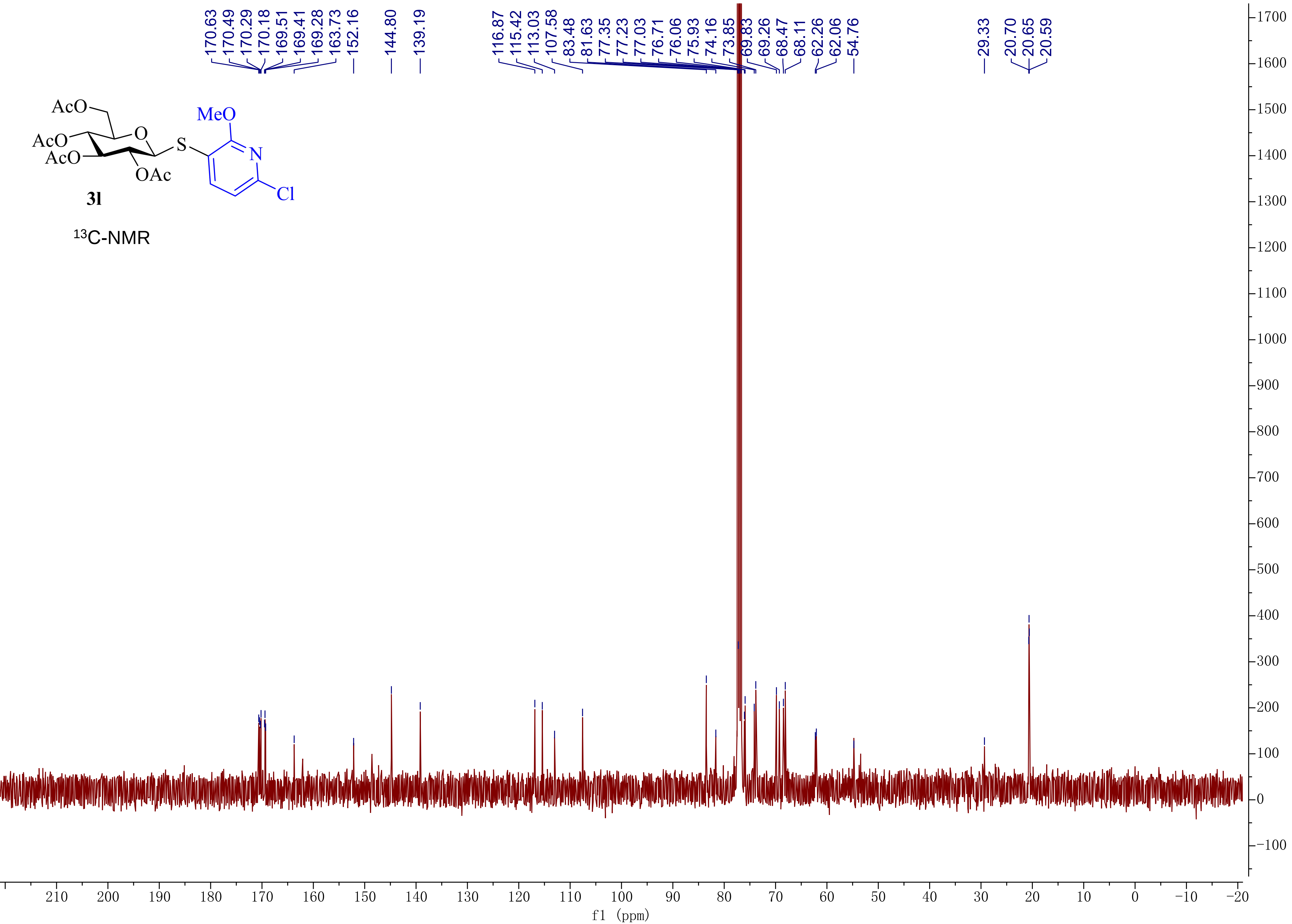

170.63
170.49
170.29
170.18
169.51
169.41
169.28
163.73
152.16
144.80
139.19
116.87
115.42
113.03
107.58
83.48
81.63
77.35
77.23
77.03
76.71
76.06
75.93
74.16
73.85
69.83
69.26
68.47
68.11
62.26
62.06
54.76
29.33
20.70
20.65
20.59
AcO
AcO
AcO
OAc
O
S
MeO
N
Cl
3l
13C-NMR
f1 (ppm)

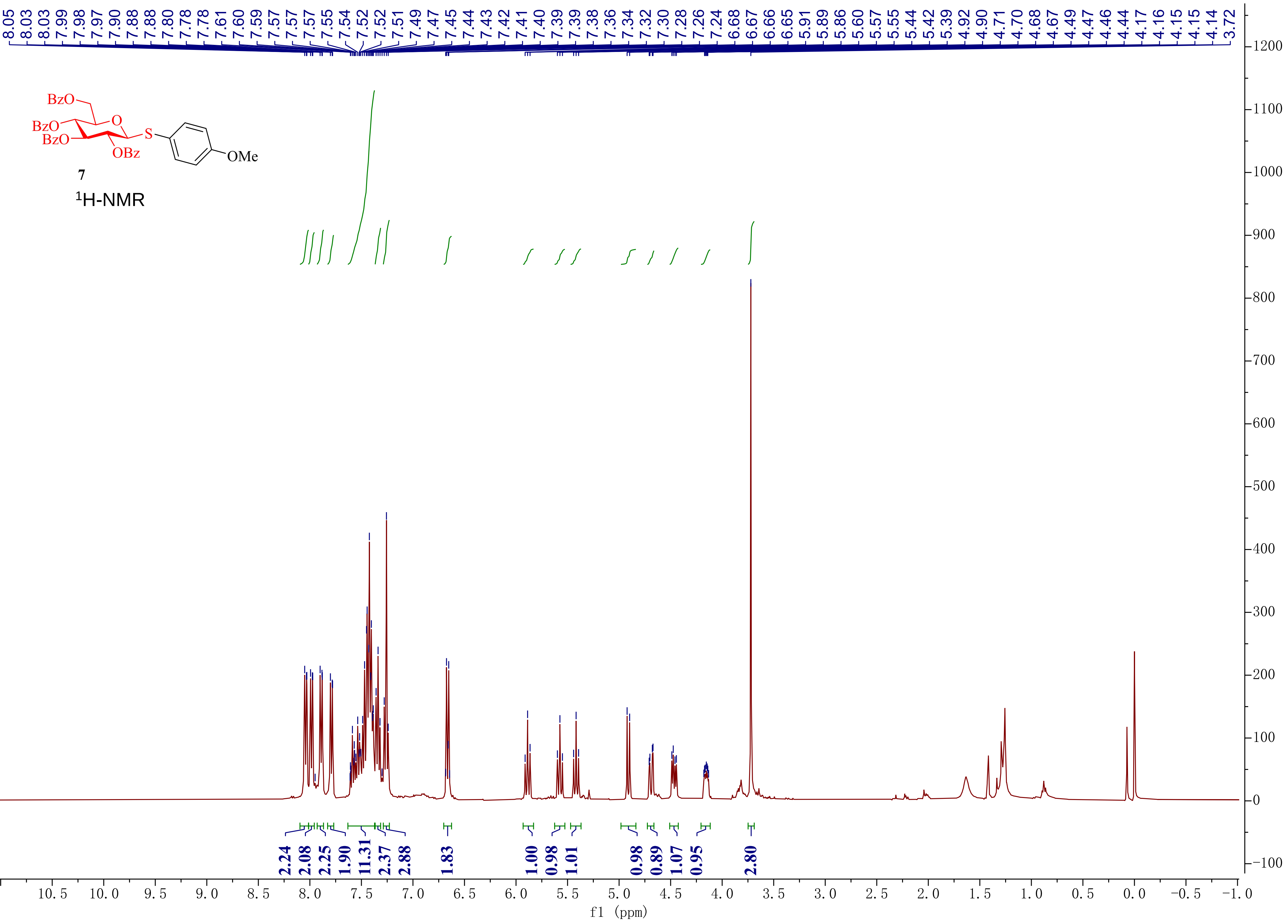
BzO
BzO
BzO
O
S
OBz
OMe
7
1H-NMR
8.05 8.03 8.03 7.99 7.98 7.97 7.90 7.88 7.88 7.80 7.78 7.78 7.61 7.60 7.59 7.57 7.57 7.55 7.54 7.52 7.52 7.51 7.49 7.47 7.45 7.44 7.43 7.42 7.41 7.40 7.39 7.39 7.38 7.36 7.34 7.32 7.30 7.28 7.26 7.24 6.68 6.67 6.66 6.65 5.91 5.89 5.86 5.60 5.57 5.55 5.44 5.42 5.39 4.92 4.90 4.71 4.70 4.68 4.67 4.49 4.47 4.46 4.44 4.17 4.16 4.15 4.15 4.14 3.72
2.24 2.08 2.25 1.90 11.31 2.37 2.88 1.83 1.00 0.98 1.01 0.98 0.89 1.07 0.95 2.80
10.5 10.0 9.5 9.0 8.5 8.0 7.5 7.0 6.5 6.0 5.5 5.0 4.5 4.0 3.5 3.0 2.5 2.0 1.5 1.0 0.5 0.0 -0.5 -1.0
f1 (ppm)
1200 1100 1000 900 800 700 600 500 400 300 200 100 0 -100

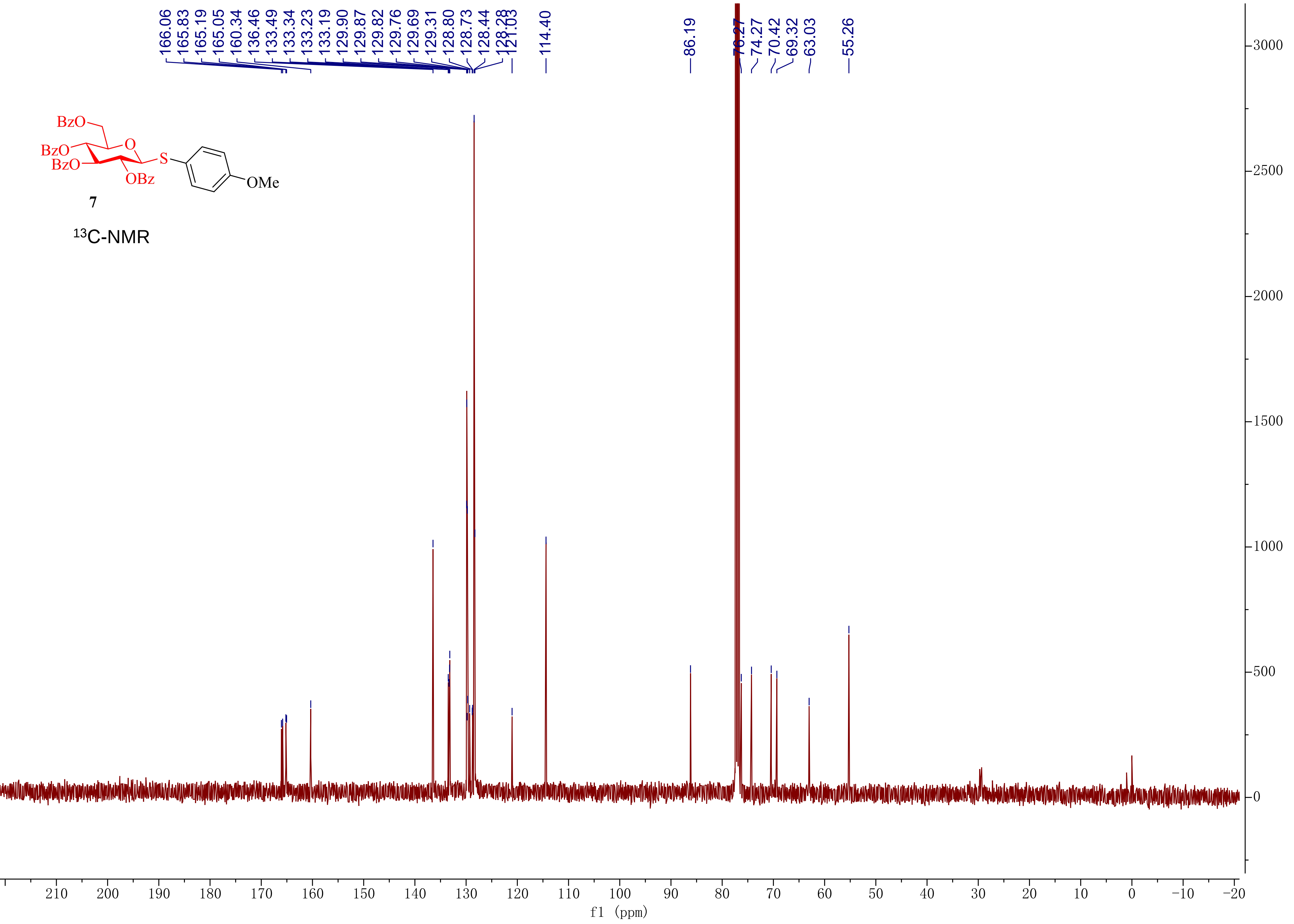
166.06
165.83
165.19
165.05
160.34
136.46
133.49
133.34
133.23
133.19
129.90
129.87
129.82
129.76
129.69
129.31
128.80
128.73
128.44
128.28
121.03
114.40
86.19
76.27
74.27
70.42
69.32
63.03
55.26
BzO
BzO
BzO
OBz
O
S
OMe
7
13C-NMR
f1 (ppm)

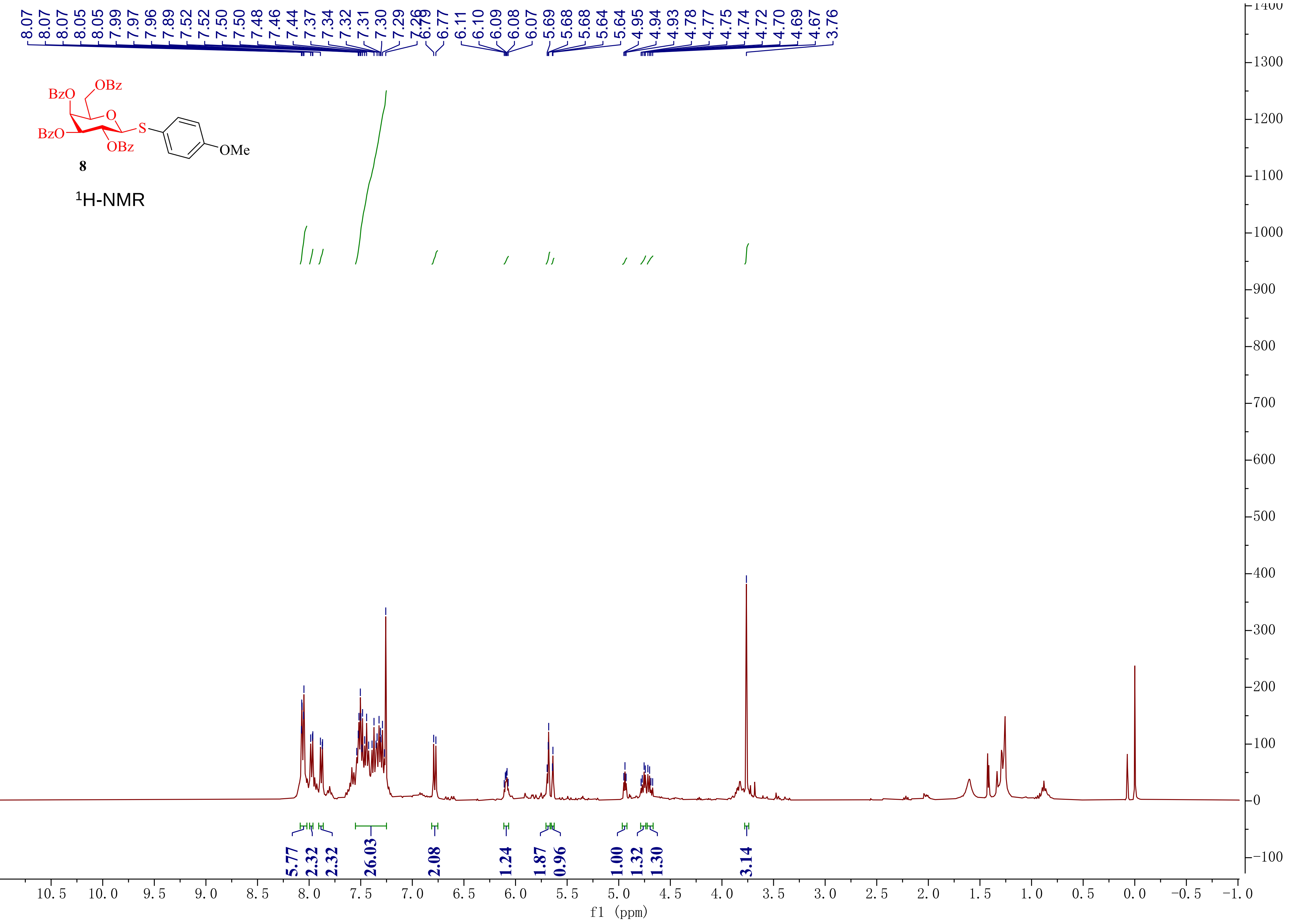
BzO
OBz
O
S
BzO
OBz
OMe
8
1H-NMR
8.07 8.07 8.07 8.05 8.05 7.99 7.97 7.96 7.89 7.52 7.52 7.50 7.50 7.48 7.46 7.44 7.37 7.34 7.32 7.31 7.30 7.29 7.26 6.79 6.77 6.11 6.10 6.09 6.08 6.07 5.69 5.68 5.68 5.64 5.64 4.95 4.94 4.93 4.78 4.77 4.75 4.74 4.72 4.70 4.69 4.67 3.76
5.77 2.32 2.32 26.03 2.08 1.24 1.87 0.96 1.00 1.32 1.30 3.14
f1 (ppm)

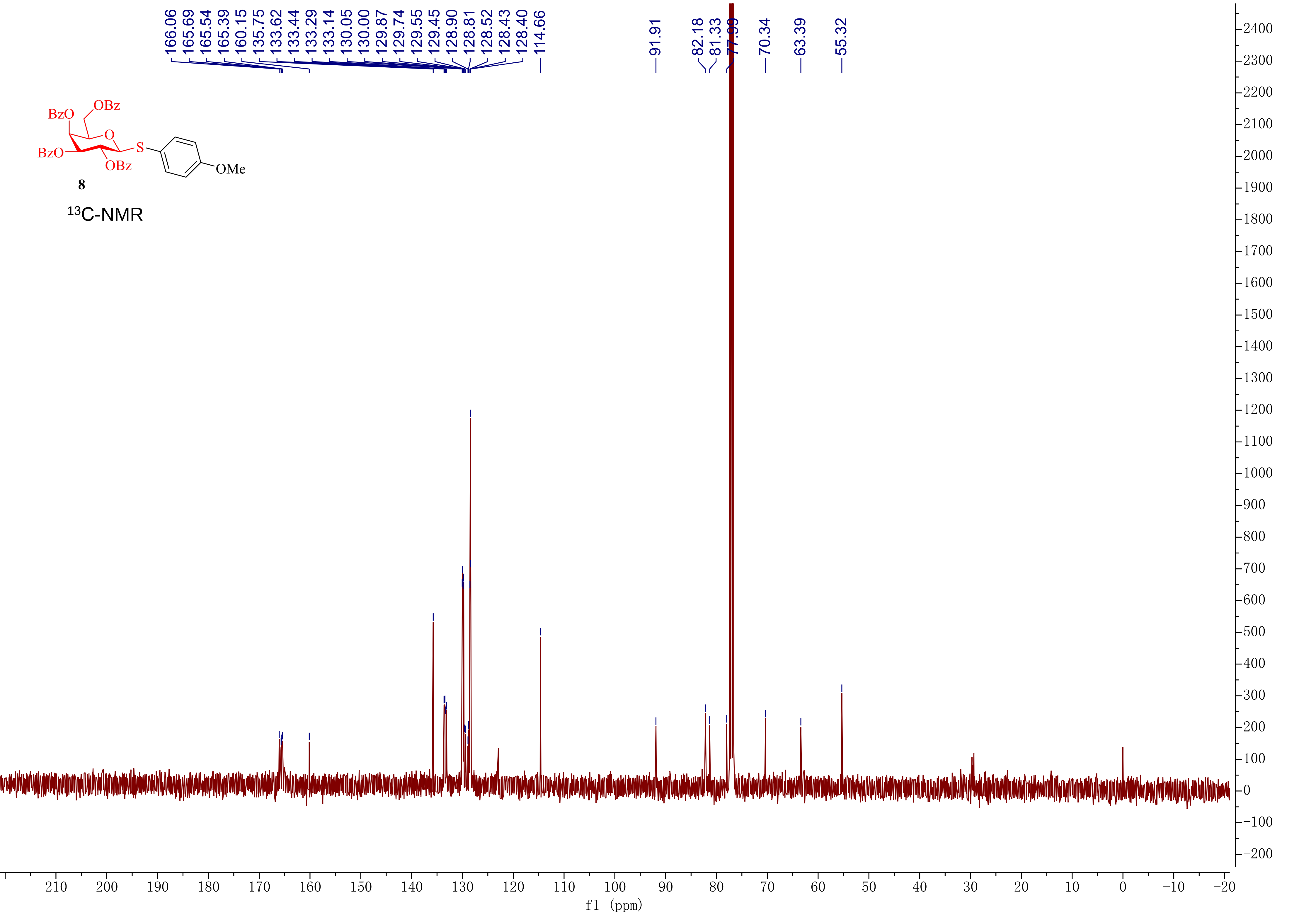
166.06
165.69
165.54
165.39
160.15
135.75
133.62
133.44
133.29
133.14
130.05
130.00
129.87
129.74
129.55
129.45
128.90
128.81
128.52
128.43
128.40
114.66
91.91
82.18
81.33
77.99
70.34
63.39
55.32
BzO
OBz
O
BzO
OBz
S
OMe
8
^{13}C-NMR
f1 (ppm)

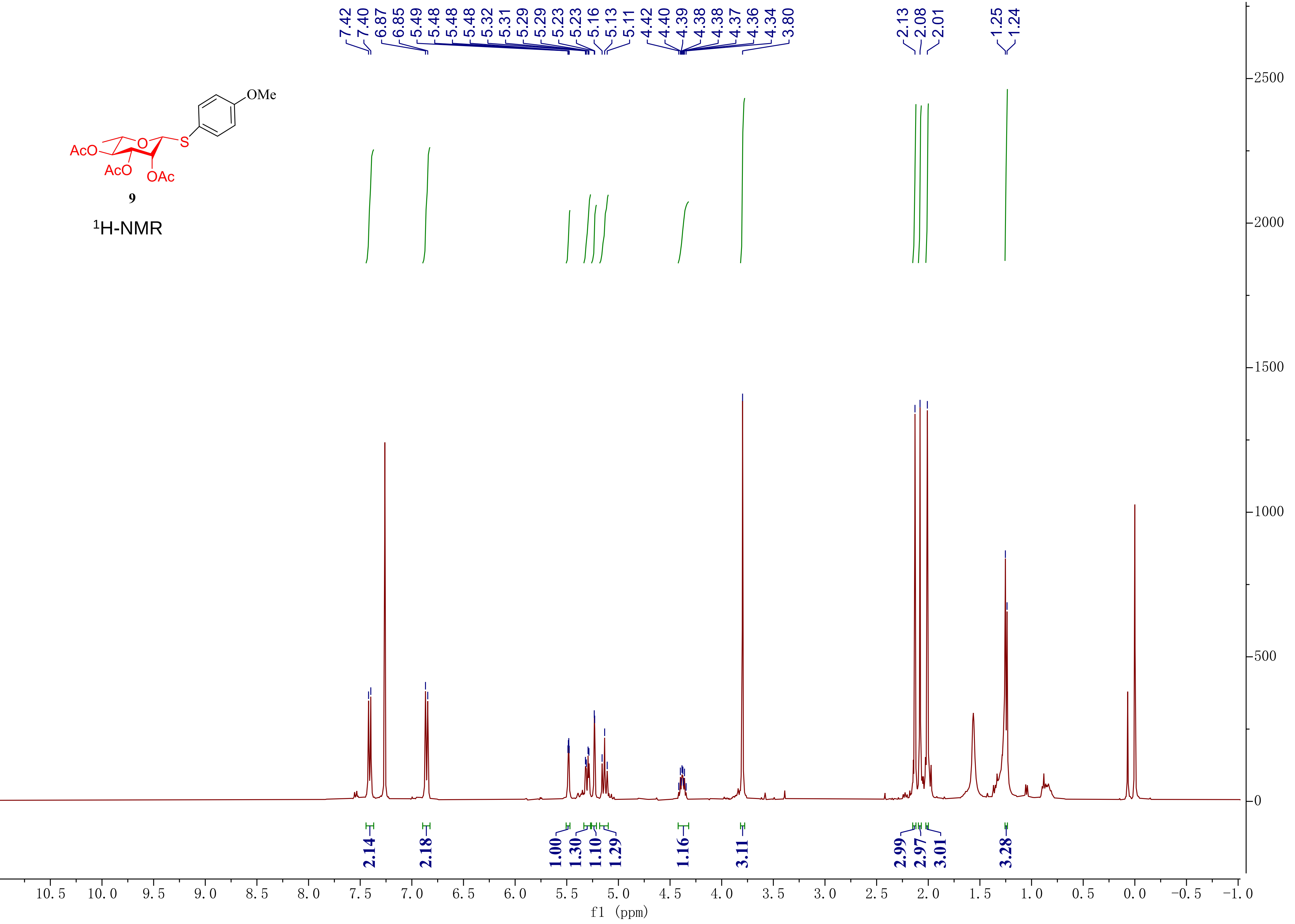

7.42
7.40
6.87
6.85
5.49
5.48
5.48
5.48
5.32
5.31
5.29
5.29
5.23
5.23
5.16
5.13
5.11
4.42
4.40
4.39
4.38
4.38
4.37
4.36
4.34
3.80
2.13
2.08
2.01
1.25
1.24
OMe
AcO
O
S
AcO
OAc
9
¹H-NMR
2.14
2.18
1.00
1.30
1.10
1.29
1.16
3.11
2.99
2.97
3.01
3.28
f1 (ppm)

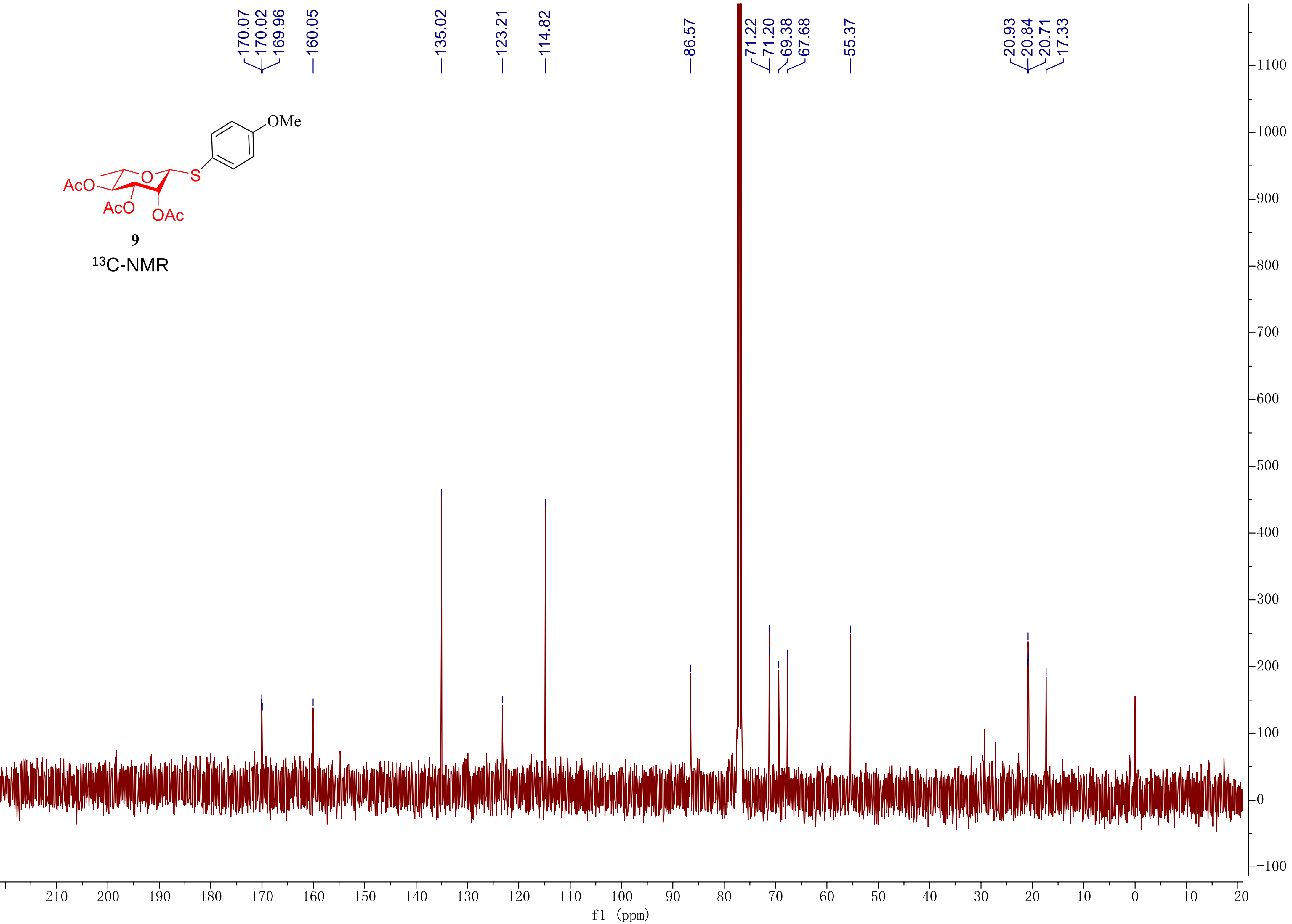
170.07
170.02
169.96
160.05
135.02
123.21
114.82
86.57
71.22
71.20
69.38
67.68
55.37
20.93
20.84
20.71
17.33
OMe
AcO
O
S
AcO
OAc
9
^{13}C-NMR
f1 (ppm)

F:\wamgzhen-1004 01/06/25 12:40:50

wamgzhen-1004 #14 RT: 0.06 AV: 1 NL: 2.04E5
T: FTMS + p ESI Full ms [150.0000-1500.0000]

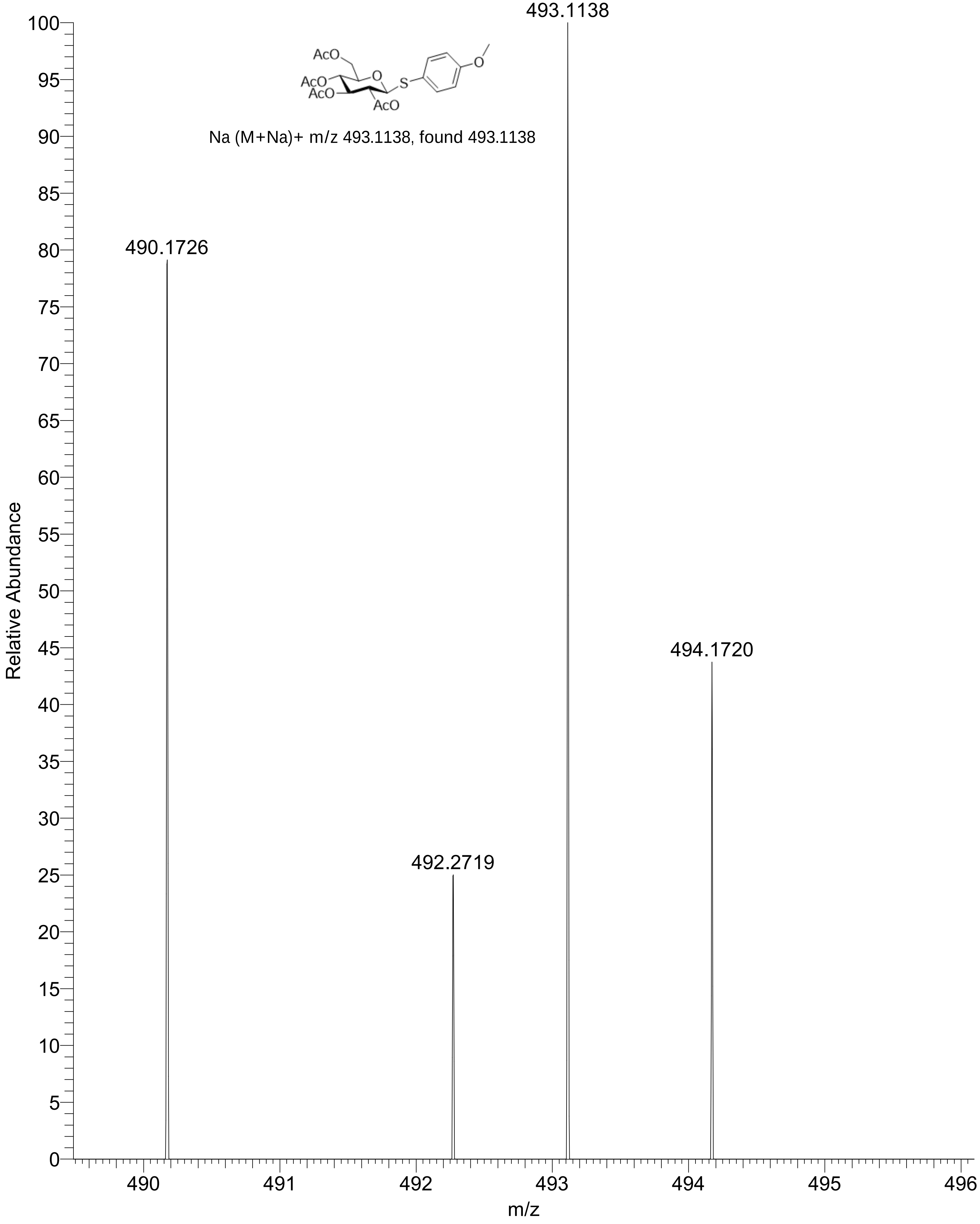

wamgzhen-1007 #34 RT: 0.15 AV: 1 NL: 1.36E6
T: FTMS + p ESI Full ms [150.0000-1500.0000]

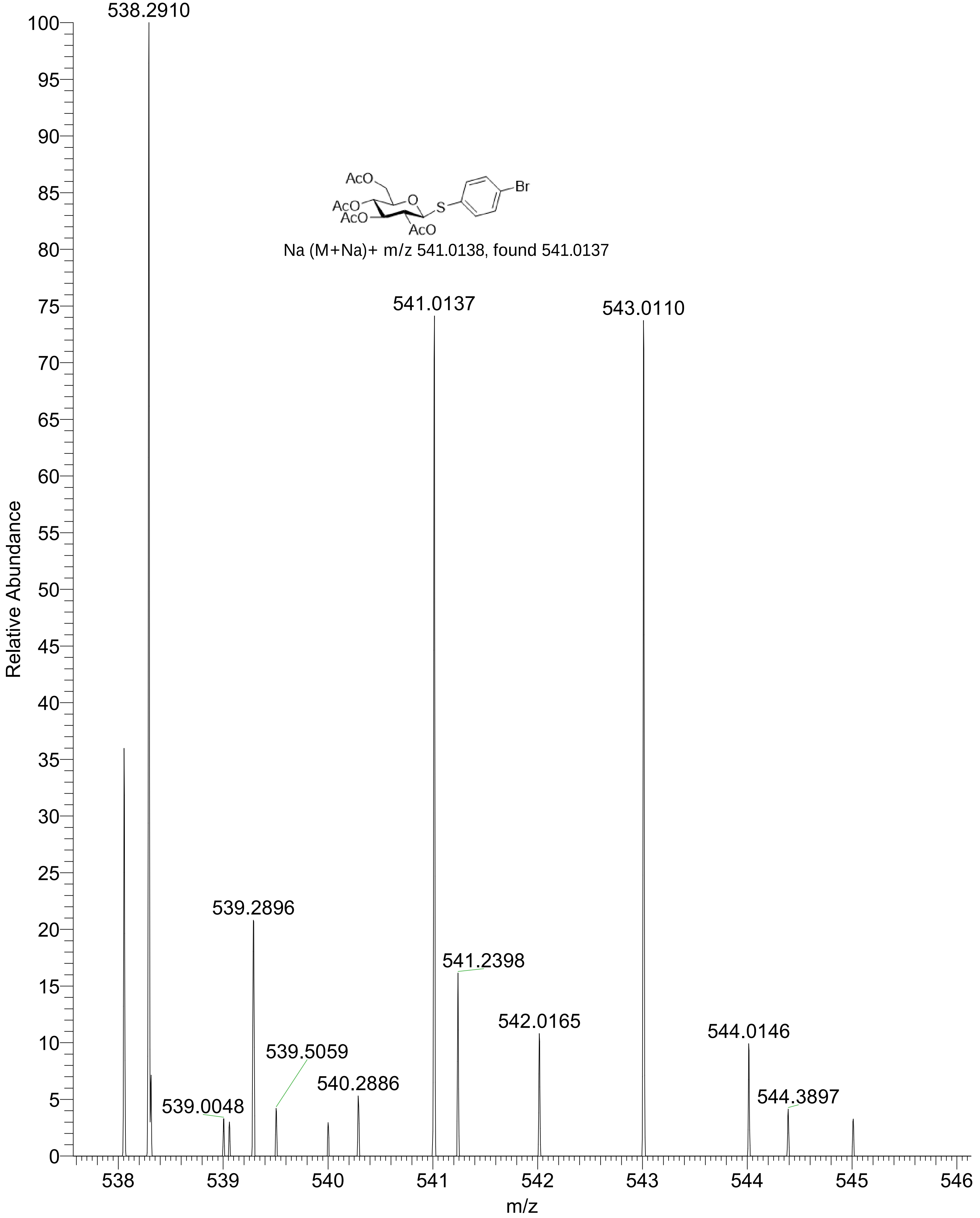

wamgzhen-1010 #44 RT: 0.19 AV: 1 NL: 2.22E5
T: FTMS + p ESI Full ms [150.0000-1500.0000]

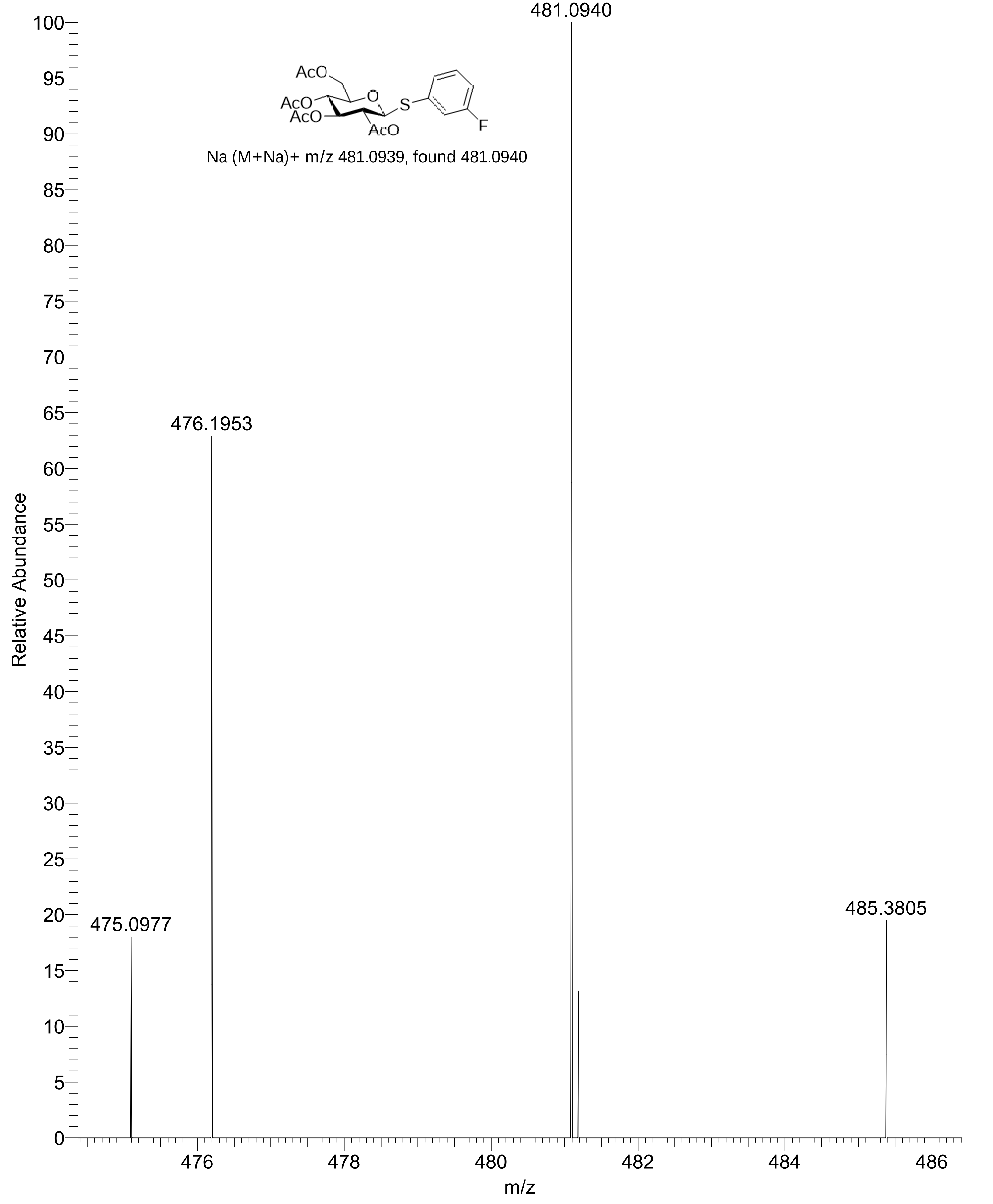

wamgzhen-1005 #15 RT: 0.07 AV: 1 NL: 2.49E5
T: FTMS + p ESI Full ms [150.0000-1500.0000]

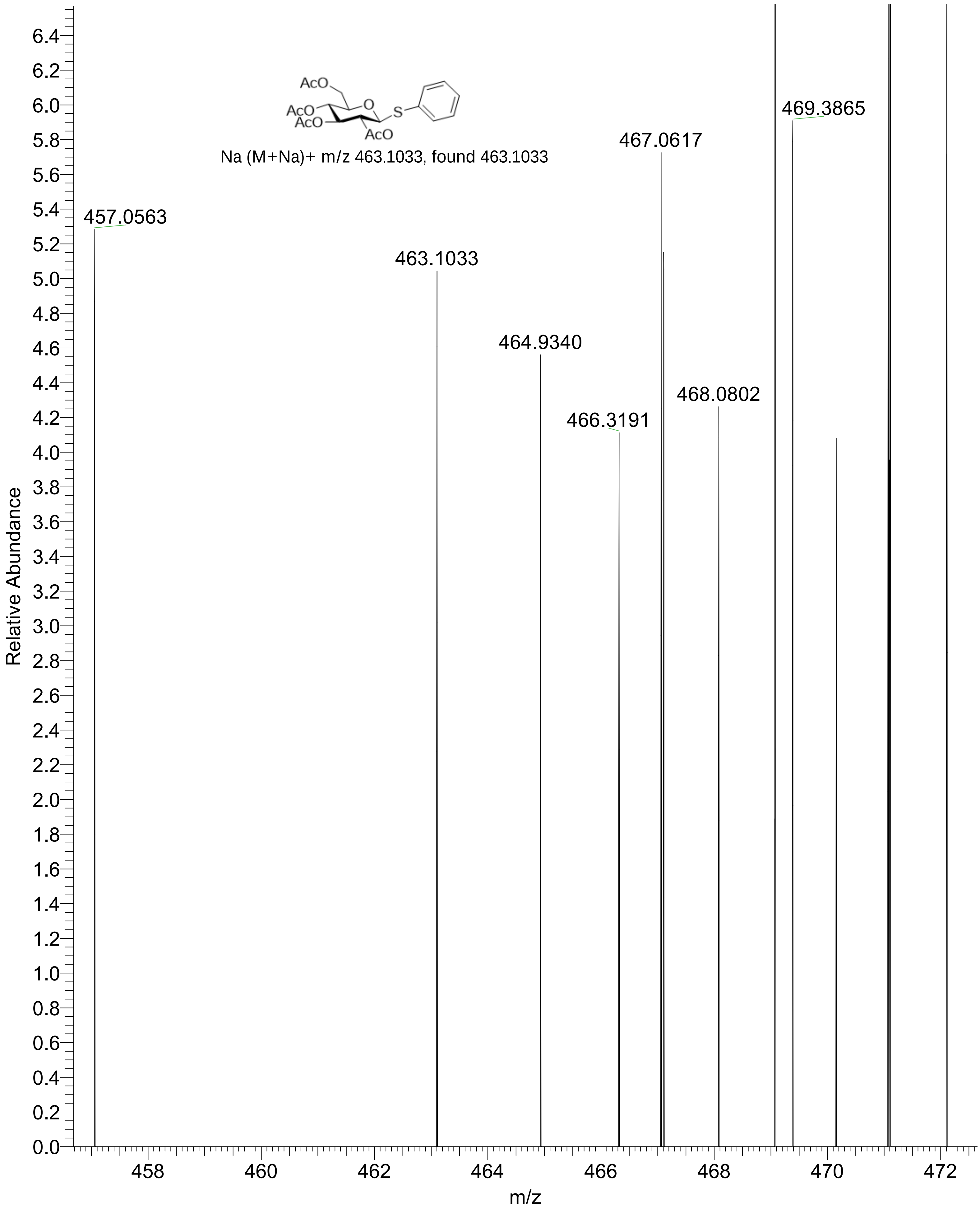

F:\wamgzhen-1011 01/06/25 12:56:12

wamgzhen-1011 #20 RT: 0.09 AV: 1 NL: 6.31E5
T: FTMS + p ESI Full ms [150.0000-1500.0000]

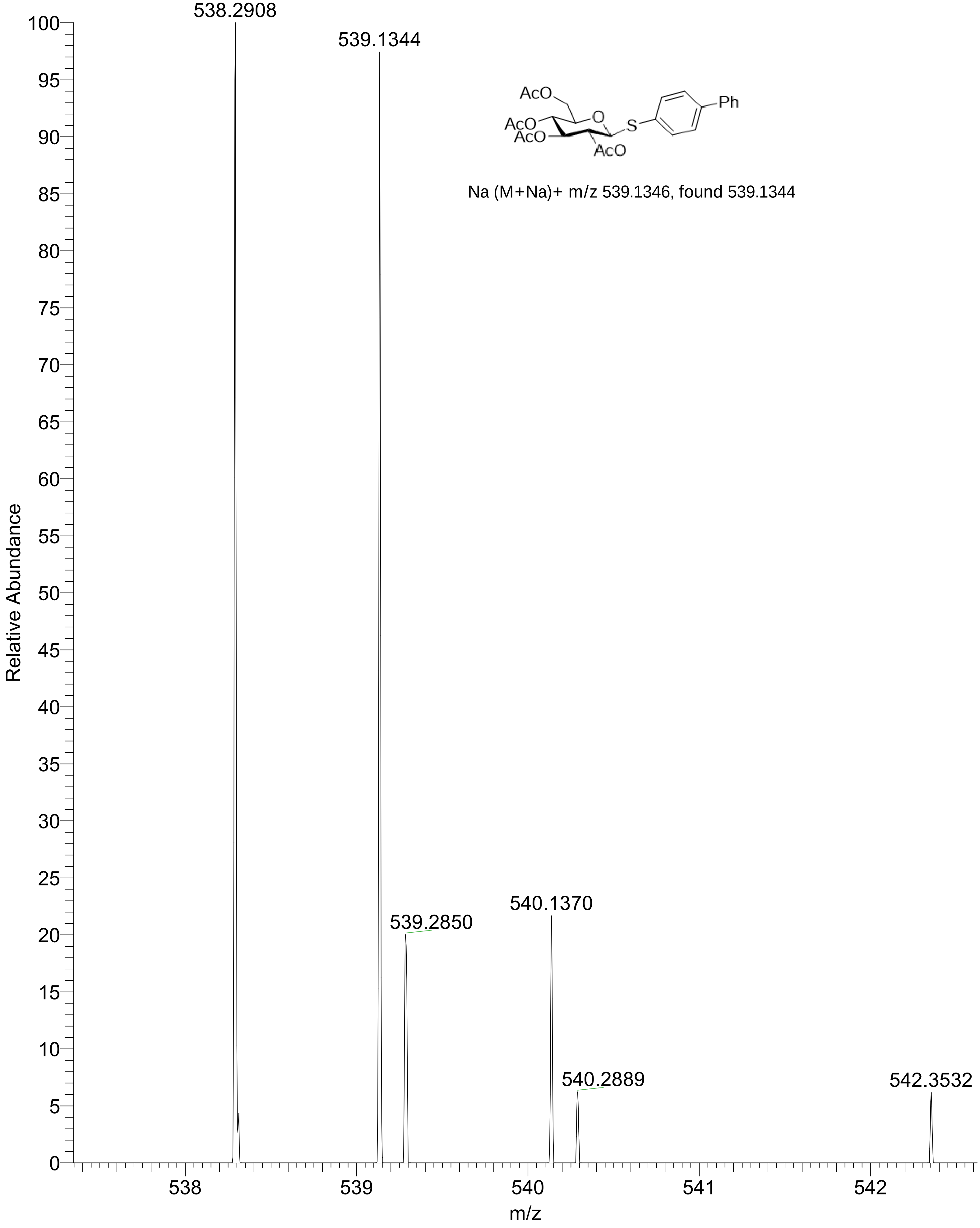

wamgzhen-1006 #40 RT: 0.18 AV: 1 NL: 6.82E6
T: FTMS + p ESI Full ms [150.0000-1500.0000]

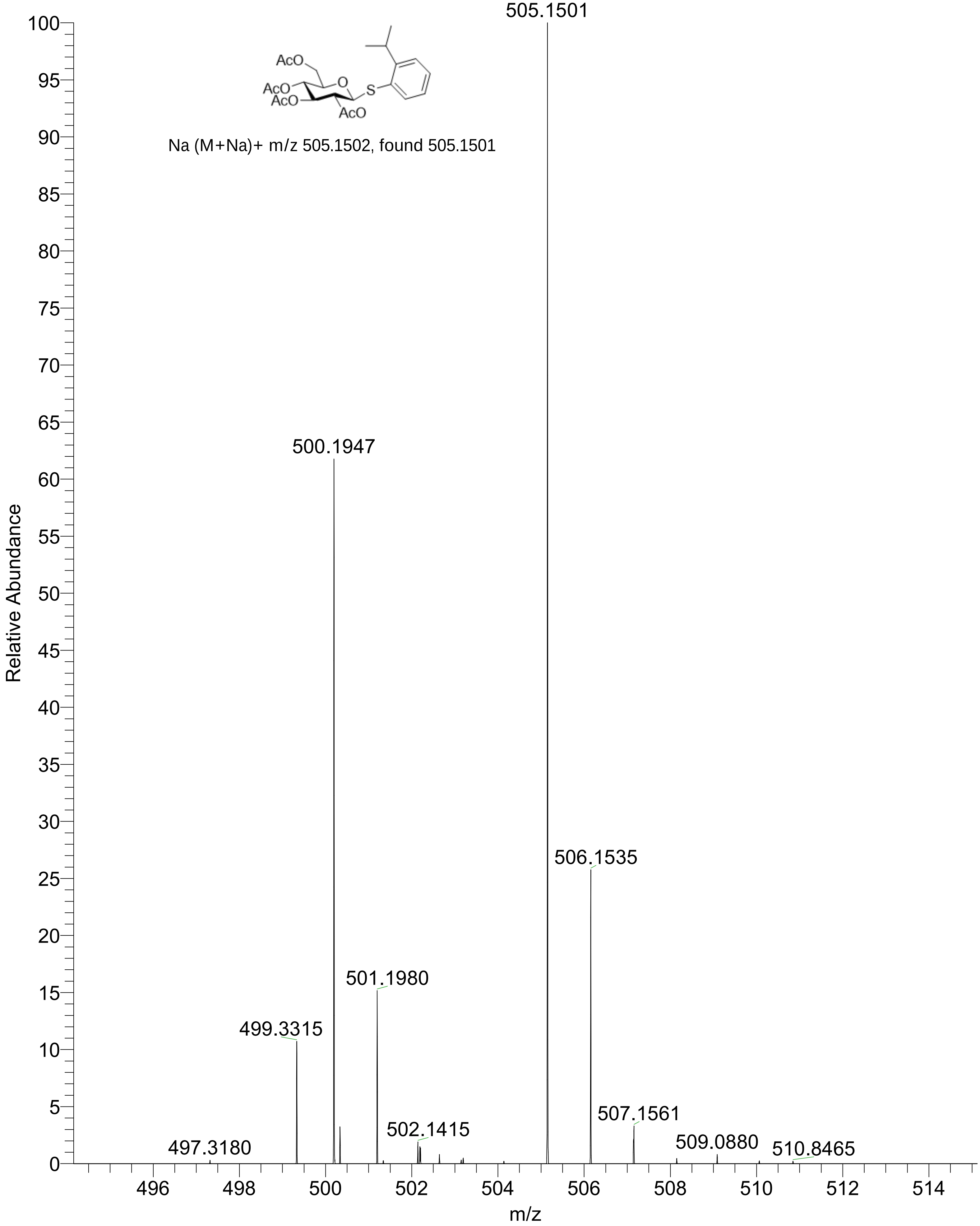

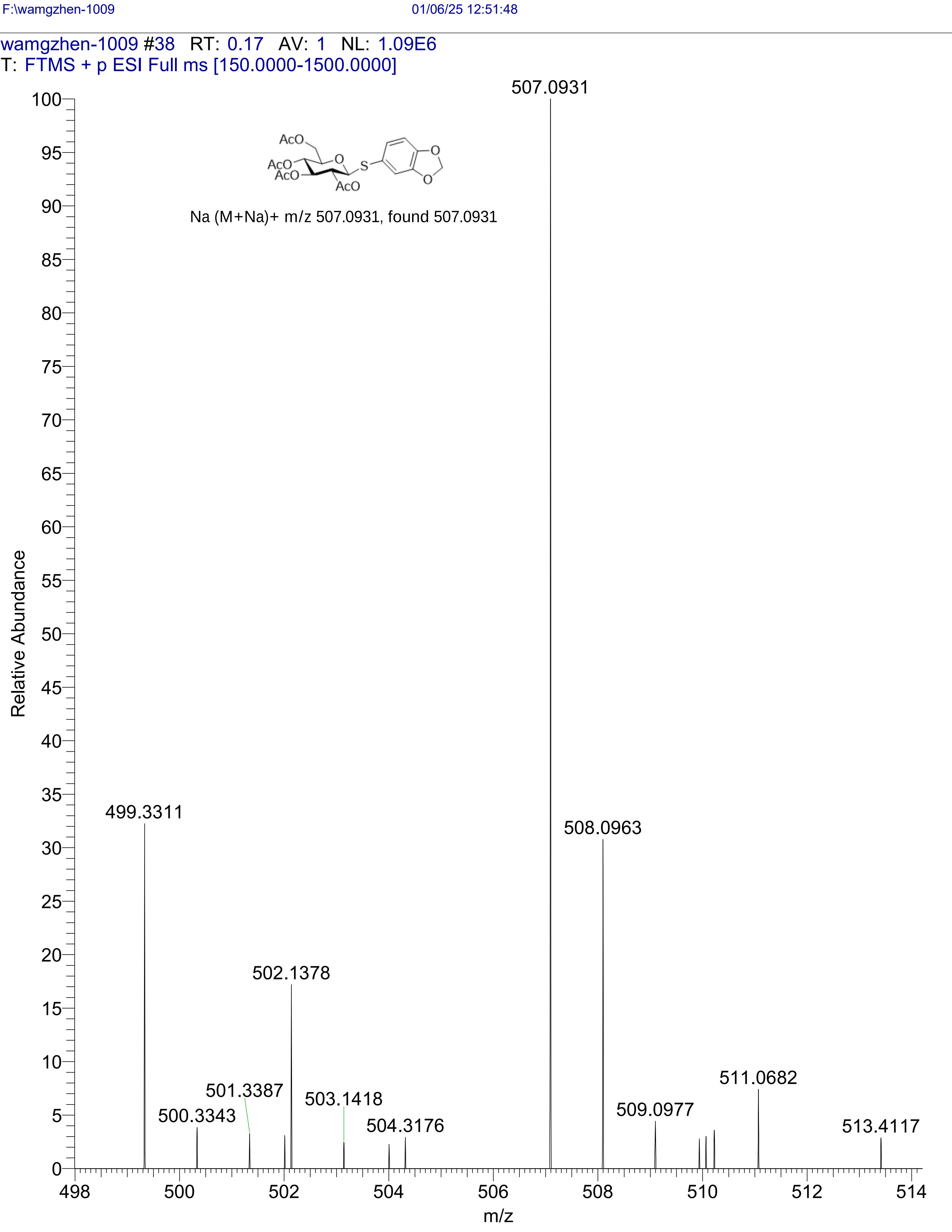

F:\wamgzhen-1009
01/06/25 12:51:48
wamgzhen-1009 #38 RT: 0.17 AV: 1 NL: 1.09E6
T: FTMS + p ESI Full ms [150.0000-1500.0000]
AcO
AcO
AcO
AcO
O
S
O
O
Na (M+Na)+ m/z 507.0931, found 507.0931
507.0931
499.3311
508.0963
502.1378
501.3387
500.3343
503.1418
504.3176
509.0977
511.0682
513.4117
Relative Abundance
m/z

F:\wamgzhen-1008 01/06/25 12:49:37

wamgzhen-1008 #21 RT: 0.09 AV: 1 NL: 7.70E5
T: FTMS + p ESI Full ms [150.0000-1500.0000]

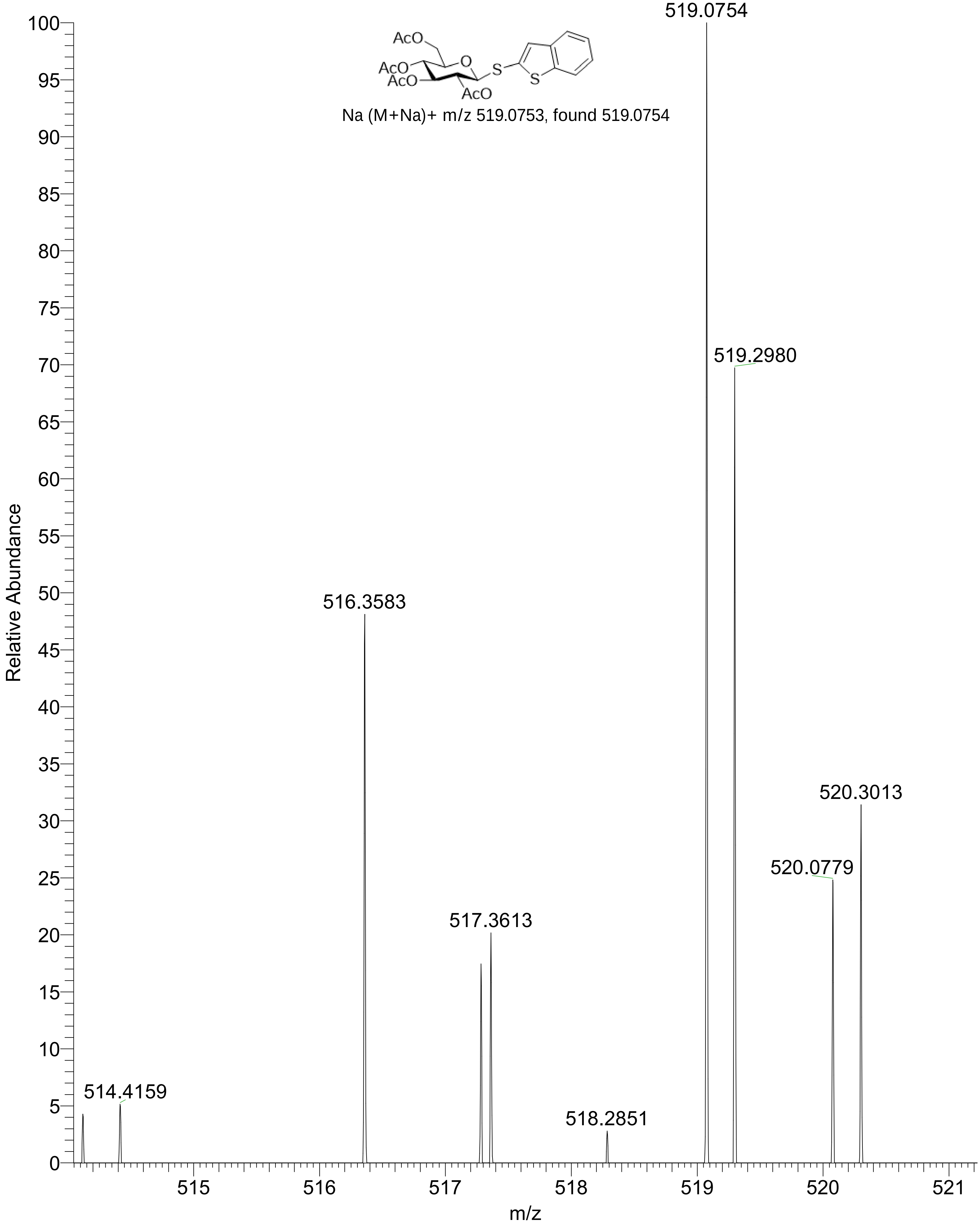

wamgzhen-1009 #38 RT: 0.17 AV: 1 NL: 1.09E6
T: FTMS + p ESI Full ms [150.0000-1500.0000]

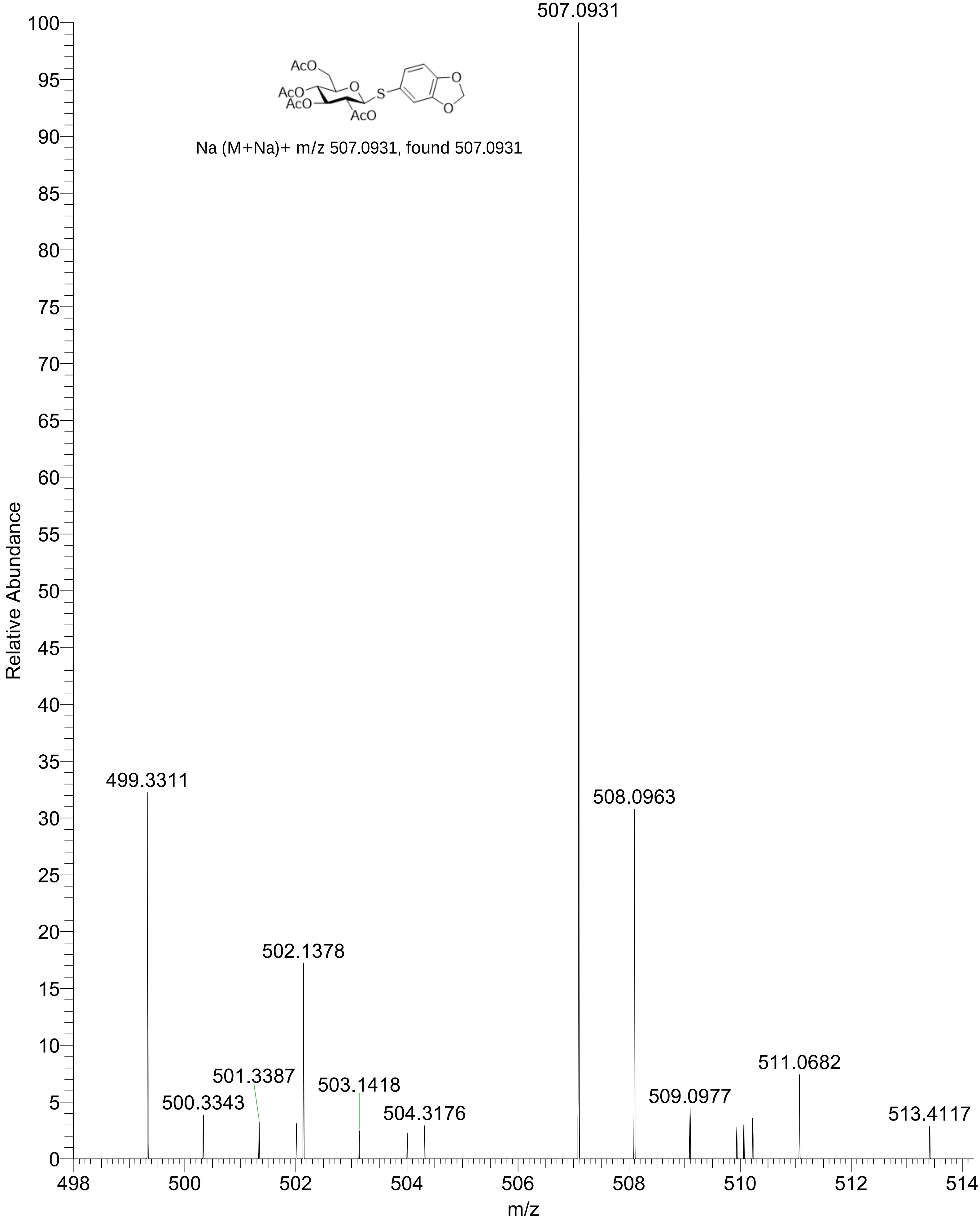

F:\wamgzhen-1012 01/06/25 12:58:25

wamgzhen-1012 #36 RT: 0.16 AV: 1 NL: 1.41E5
T: FTMS + p ESI Full ms [150.0000-1500.0000]

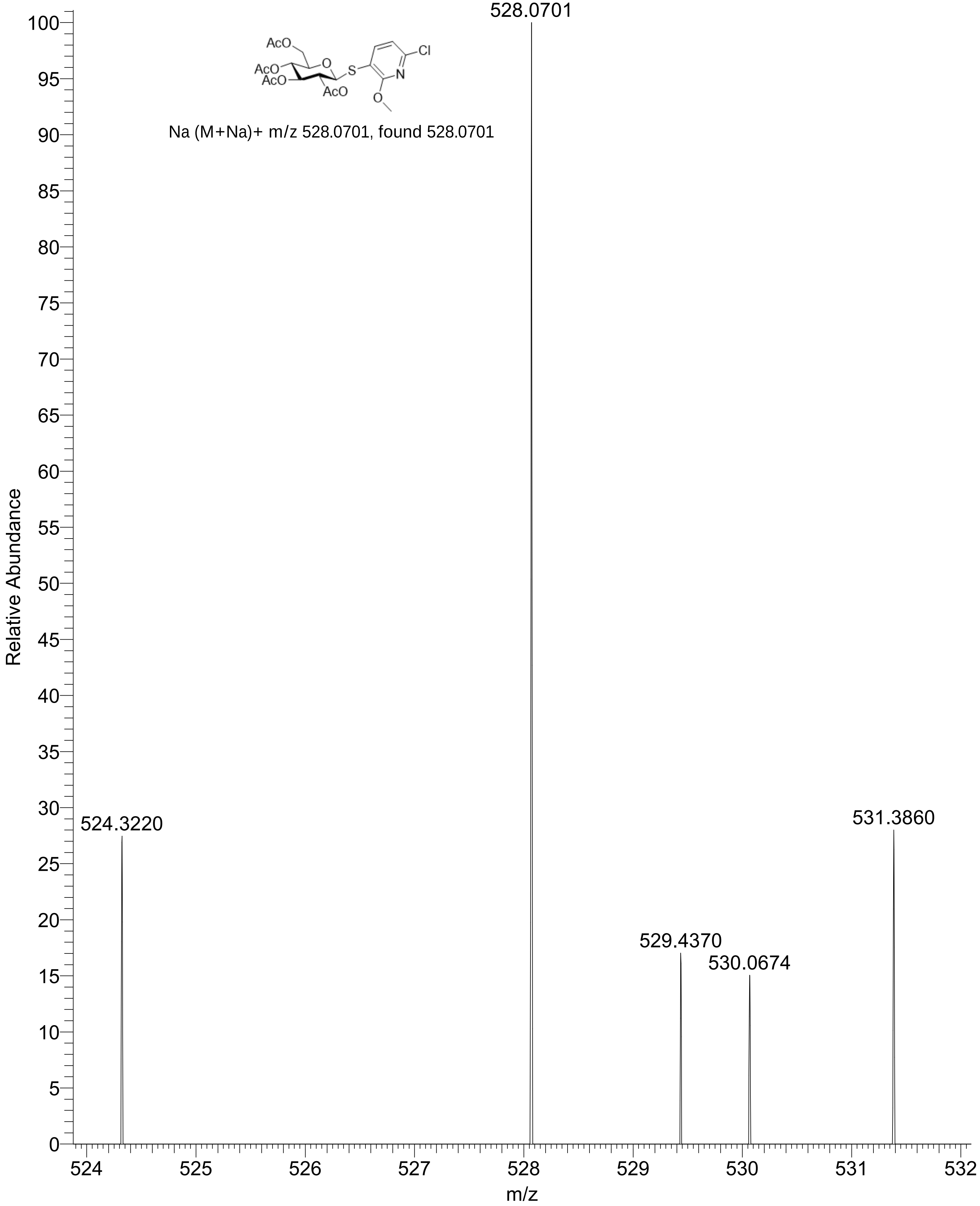

wamgzhen-1001 #25 RT: 0.11 AV: 1 NL: 1.13E7
T: FTMS + p ESI Full ms [150.0000-1500.0000]

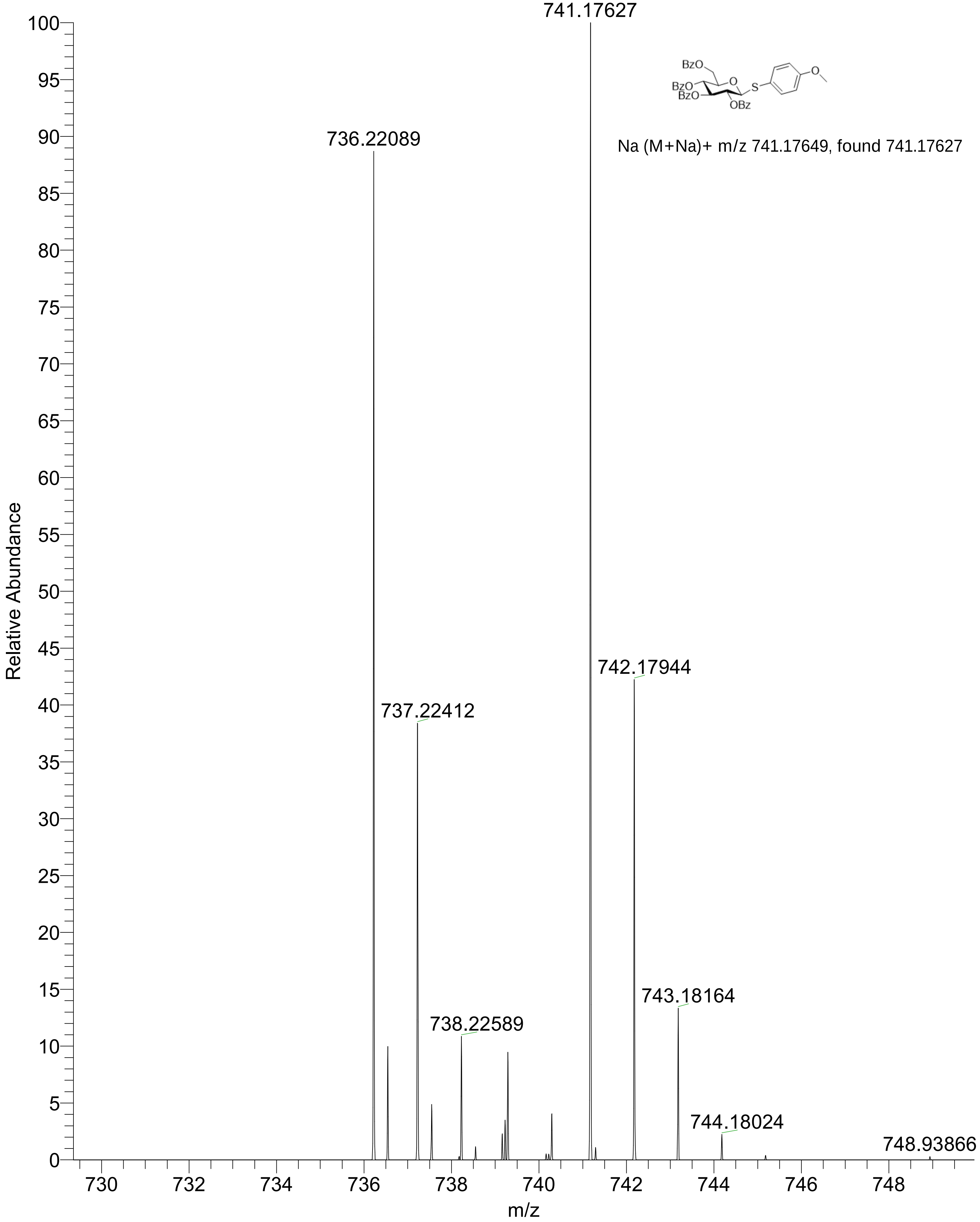

F:\wamgzhen-1002 01/06/25 12:36:27

wamgzhen-1002 #5 RT: 0.02 AV: 1 NL: 5.67E5
T: FTMS + p ESI Full ms [150.0000-1500.0000]

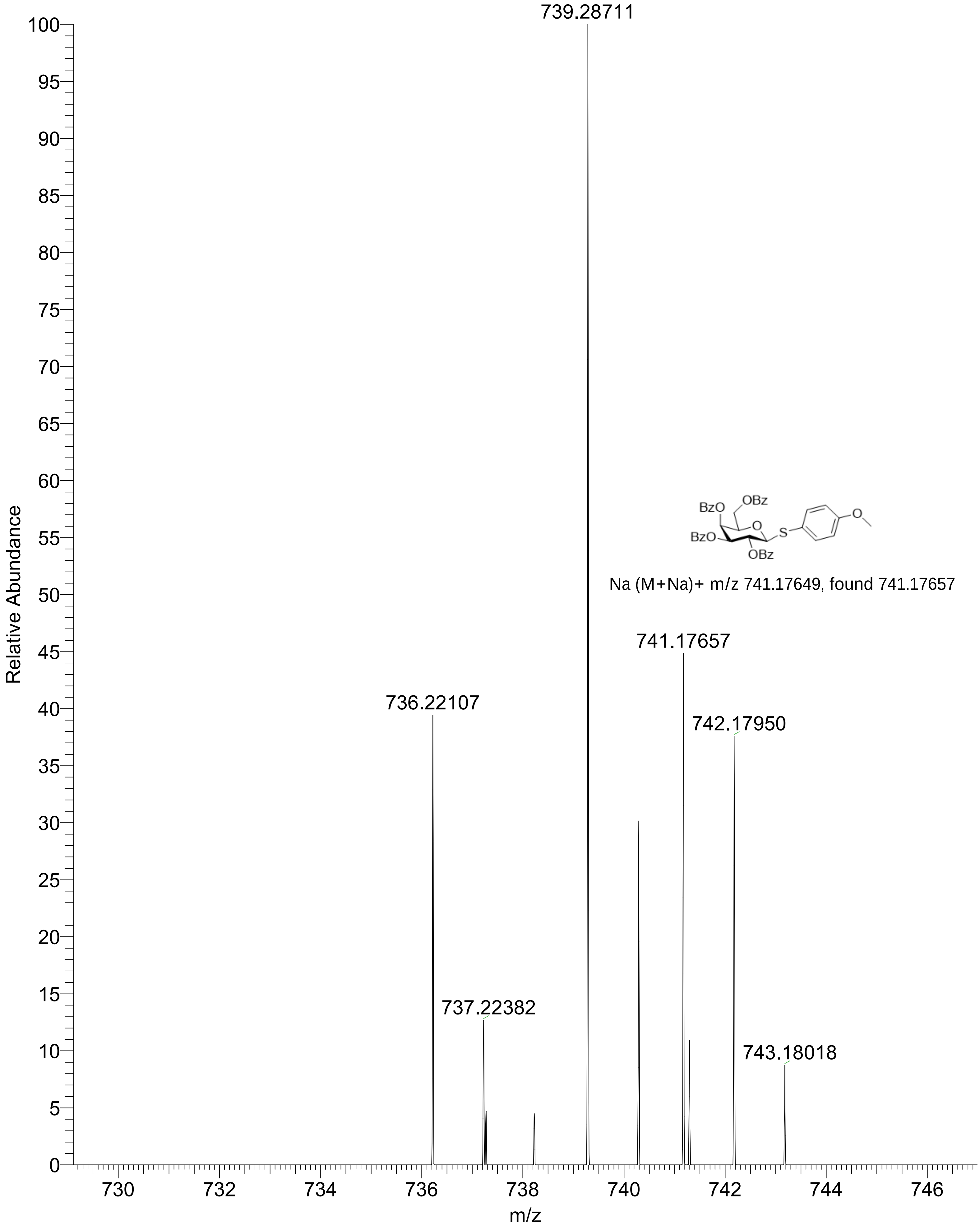

wamgzhen-1003 #21 RT: 0.09 AV: 1 NL: 1.40E6
T: FTMS + p ESI Full ms [150.0000-1500.0000]

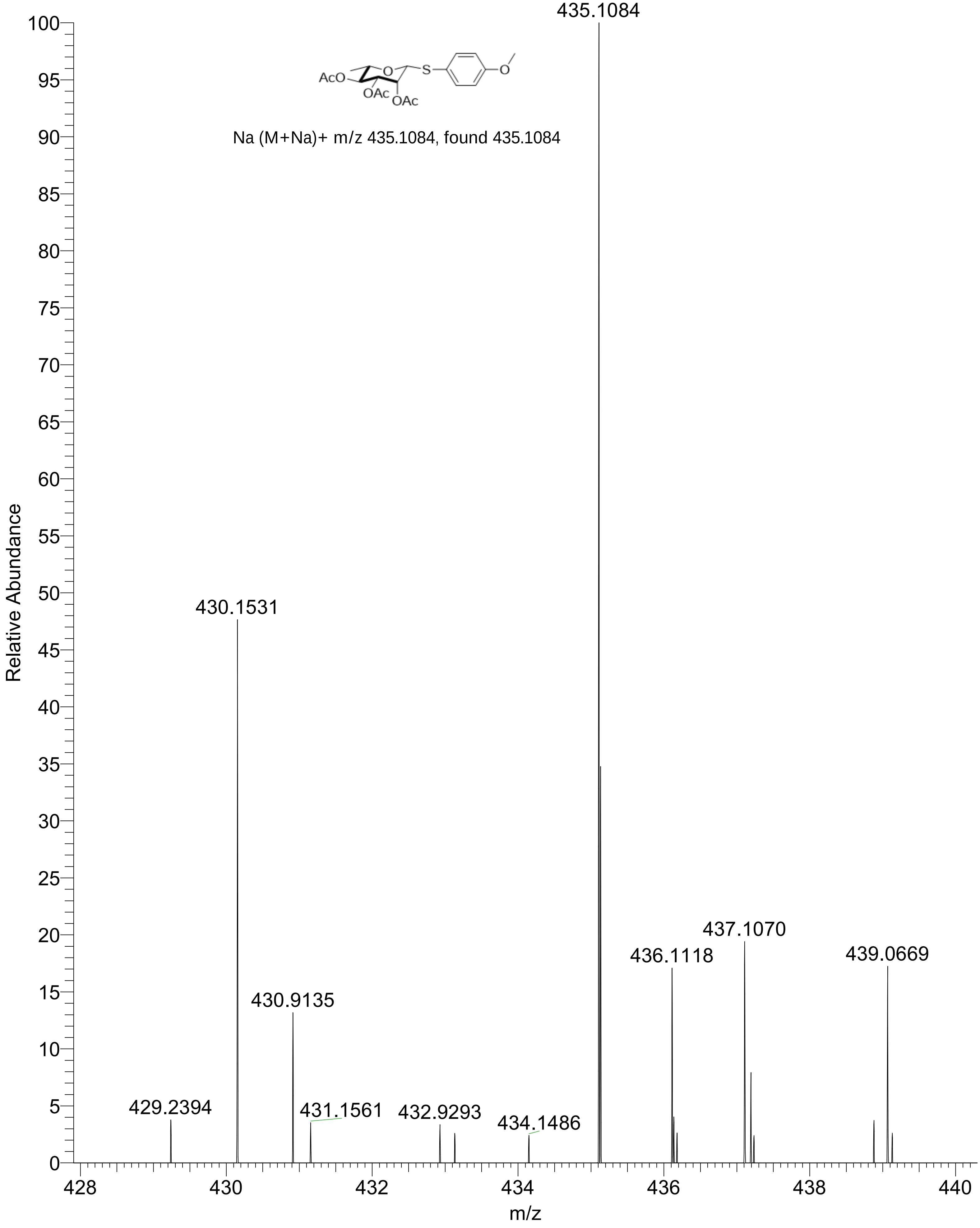

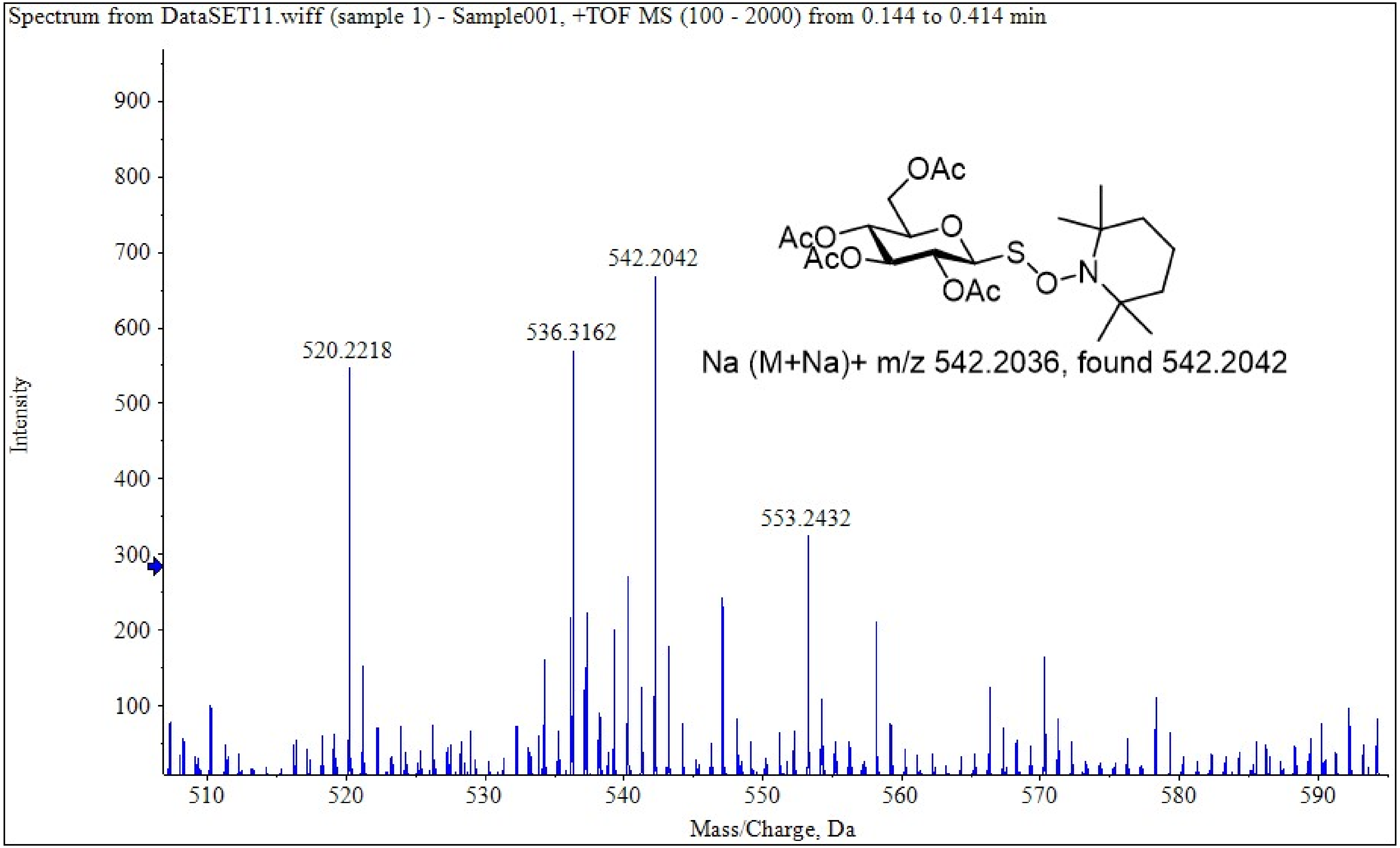

2025/2/20 12:36:50

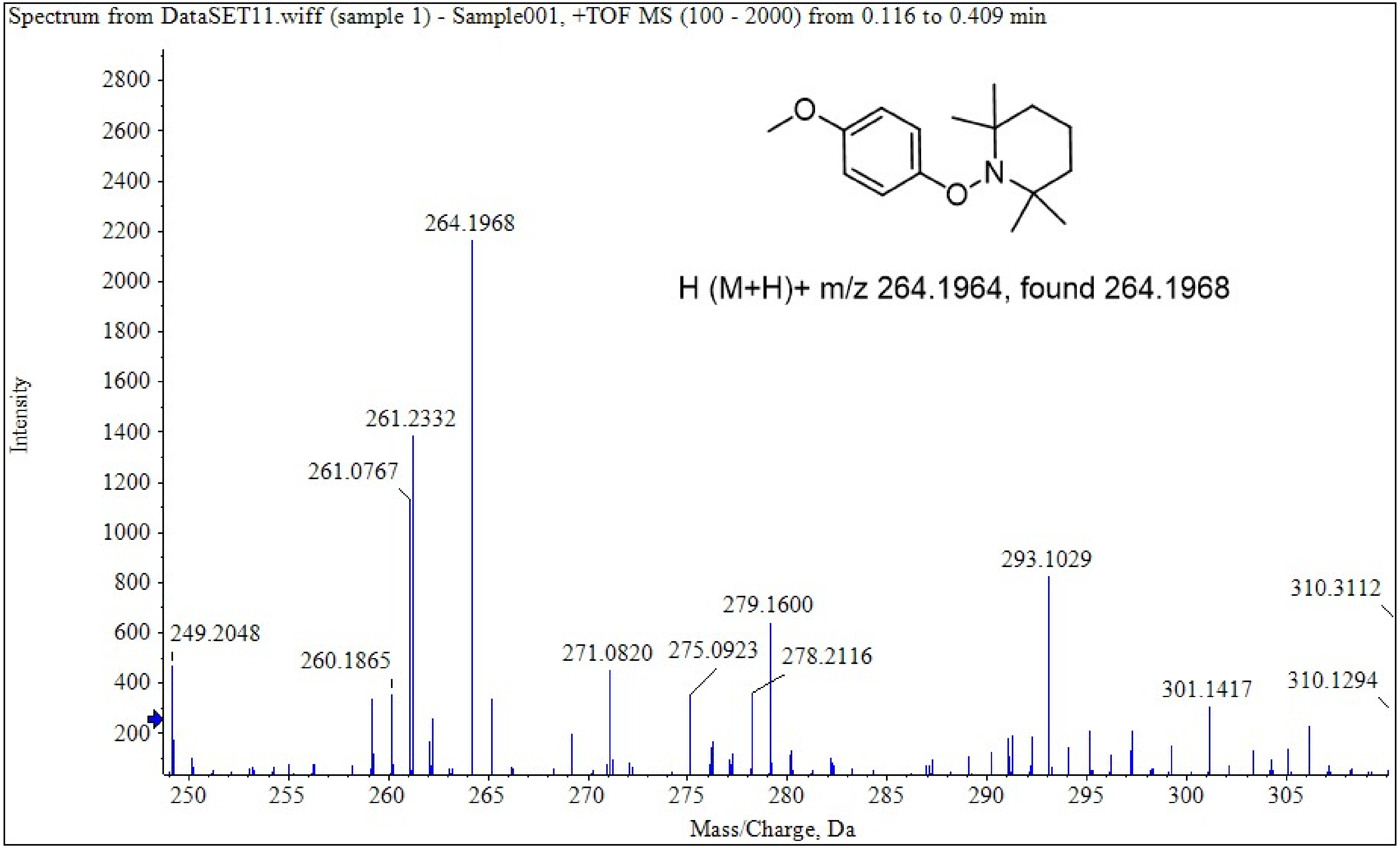

2025/2/20 12:27:46
